# Targeted and Random Mutagenesis of *Ehrlichia chaffeensis* for the Identification of Genes Required for *In vivo* Infection

**DOI:** 10.1371/journal.ppat.1003171

**Published:** 2013-02-14

**Authors:** Chuanmin Cheng, Arathy D. S. Nair, Vijaya V. Indukuri, Shanzhong Gong, Roderick F. Felsheim, Deborah Jaworski, Ulrike G. Munderloh, Roman R. Ganta

**Affiliations:** 1 Department of Diagnostic Medicine/Pathobiology, College of Veterinary Medicine, Kansas State University, Manhattan, Kansas, United States of America; 2 Department of Entomology, University of Minnesota, St. Paul, Minnesota, United States of America; 3 Department of Entomology and Plant Pathology, Oklahoma State University, Noble Research Center, Stillwater, Oklahoma, United States of America; Yale University School of Medicine, United States of America

## Abstract

*Ehrlichia chaffeensis* is a tick transmitted pathogen responsible for the disease human monocytic ehrlichiosis. Research to elucidate gene function in rickettsial pathogens is limited by the lack of genetic manipulation methods. Mutational analysis was performed, targeting to specific and random insertion sites within the bacterium's genome. Targeted mutagenesis at six genomic locations by homologous recombination and mobile group II intron-based methods led to the consistent identification of mutants in two genes and in one intergenic site; the mutants persisted in culture for 8 days. Three independent experiments using Himar1 transposon mutagenesis of *E. chaffeensis* resulted in the identification of multiple mutants; these mutants grew continuously in macrophage and tick cell lines. Nine mutations were confirmed by sequence analysis. Six insertions were located within non-coding regions and three were present in the coding regions of three transcriptionally active genes. The intragenic mutations prevented transcription of all three genes. Transposon mutants containing a pool of five different insertions were assessed for their ability to infect deer and subsequent acquisition by *Amblyomma americanum* ticks, the natural reservoir and vector, respectively. Three of the five mutants with insertions into non-coding regions grew well in deer. Transposition into a differentially expressed hypothetical gene, Ech_0379, and at 18 nucleotides downstream to Ech_0230 gene coding sequence resulted in the inhibition of growth in deer, which is further evidenced by their failed acquisition by ticks. Similarly, a mutation into the coding region of ECH_0660 gene inhibited the *in vivo* growth in deer. This is the first study evaluating targeted and random mutagenesis in *E. chaffeensis*, and the first to report the generation of stable mutants in this obligate intracellular bacterium. We further demonstrate that *in vitro* mutagenesis coupled with *in vivo* infection assessment is a successful strategy in identifying genomic regions required for the pathogen's *in vivo* growth.

## Author Summary

The tick-transmitted bacterium, *Ehrlichia chaffeensis*, causes human monocytic ehrlichiosis, an acute febrile illness that can progress to a fatal outcome. This and other related pathogens have evolved to establish infections in vertebrate and tick hosts for completing their lifecycle. Our recent studies suggest that the pathogen's differential gene expression during growth in ticks and mammals is a major contributor for its dual host adaptation. However, the importance of the pathogen phenotype differences is best understood if we have methods to knock down protein expression from one or more genes. Creating mutations in obligate intracellular pathogens remain a challenge due to their limited survival in the extracellular environment. Here, we present evidence for multiple insertion mutations in the *E. chaffeensis* genome. Three of the nine mutations in the genome inhibiting gene expression prevented infection of deer, the natural host for the pathogen. This is the first study demonstrating the feasibility of creating mutations in an *Ehrlichia* species; and directly linking specific regions of the genome to *in vivo* infection. Methods described here allow for studies to define genes important for infectivity and ability to cause disease, and are equally important for initiating similar studies in other related emerging zoonotic pathogens.

## Introduction

*Ehrlichia chaffeensis*, an alpha-proteobacterium, is an intracellular pathogen that is transmitted through an infected Lone Star tick, *Amblyomma americanum,* to humans and several other vertebrate hosts [1]–[8]. The pathogen is responsible for causing human monocytic ehrlichiosis (HME) [6], [7], [9], [10]. The disease is characterized by an acute onset of febrile illness that can progress to a fatal outcome, particularly in immune compromised individuals [2], [11]. Clinical symptoms of the flu like illness include malaise, nausea, headache, myalgia and persistent fever. Leukopenia, thrombocytopenia, and elevated liver transaminases are common laboratory findings [9], [10], [12].

*E. chaffeensis* and related pathogens have evolved unique strategies to establish infections in both ticks and mammals in order to successfully complete their transmission cycle [13], [14]. Persistent infection throughout the developmental stages of ticks is important, as the organism cannot be transmitted transovarially to larval offspring. Our recent molecular and proteomic studies have revealed global differences in the expressed proteins of *E. chaffeensis* within different host cell environments [15]–[18]. The pathogen's differential gene expression in response to distinct cellular environments is a major contributor for its dual host adaptation and persistence [19].

Targeted or random mutagenesis is routinely employed to study gene function in bacteria that can be grown axenically. Transformation of obligate intracellular pathogens, such as *E. chaffeensis*, remains a challenge, particularly considering its limited viability in the extra-cellular environment [20]. Transformation experiments, therefore, must be conducted during a small window of time when the bacteria must remain competent to infect host cells. Secondly, as *Ehrlichia* species do not harbor plasmids, the introduced foreign DNA in the form of a plasmid or linear fragments must remain intact during the transformation and infection stages so that the encoded genes can be expressed using the bacterium's RNA polymerase complex. Finally, the choice of an antibiotic resistance cassette to be introduced into the organisms should not target antibiotics useful in treating a patient. In this study, we considered all these aspects in creating mutational methods in *E. chaffeensis*.

Homologous recombination is useful for targeting mutations to a specific genomic site, and has been successfully employed in making gene disruption mutations in several intracellular bacteria [21]–[24]. Similarly, a modified mobile group II intron (TargeTron)-based targeted mutagenesis has been proven valuable for efficient gene targeting in several Gram-negative and Gram-positive bacteria [25]–[31]. Recent studies have shown that the Himar1 transposase system is a valuable tool for transposon-mutagenesis of various bacterial organisms, including alpha-proteobacteria closely related to *E. chaffeensis*, such as *Anaplasma phagocytophilum* [32], [33].

In this study, we evaluated methods for creating targeted mutations in *E. chaffeensis* by employing both homologous recombination and TargeTron methods, and random mariner mutagenesis using the Himar1 transposase system. Six different genomic locations were assessed by targeted mutagenesis which led to the consistent identification of mutants at three genomic sites. We generated nine random transposon-mediated mutations in the *E. chaffeensis* genome, three of which disrupted the coding regions of different transcriptionally active hypothetical protein genes, and six in intergenic sites. Four of the insertions also caused loss of gene expression. We present the first evidence that insertion mutations at three sites within the *E. chaffeensis* genome abolished the growth of the organism in its natural host.

## Results

### Antibiotics inhibitory to *E. chaffeensis* growth

We evaluated the ability of spectinomycin, rifampin, chloramphenicol, gentamicin, and kanamycin and ampicillin to inhibit the growth of *E. chaffeensi*s in the canine macrophage cell line, DH82. With the exception of ampicillin, all of the antibiotics inhibited the growth of the organism. The minimum concentrations of antibiotics required for 100% growth inhibition by day 7 varied, and they were 100 µg/ml for spectinomycin, 0.1 µg/ml for rifampin, 80 µg/ml for gentamicin, 4 µg/ml for chloramphenicol and 50 µg/ml for kanamycin. We opted to use antibiotic resistance gene cassettes against spectinomycin (which also confers resistance to streptomycin [32] and chloramphenicol for assessing the growth of *E. chaffeensis* following the introduction of insertion mutations in the organism, as they were proven valuable in similar studies in other intracellular bacteria of the genera *Anaplasma, Coxiella* and *Rickettsia* [32], [34], [35]. A gentamicin resistance cassette was also used in this study, but as the results for this cassette were similar to the chloramphenicol acetyl transferase (CAT) gene cassette (described below), the use of this cassette was not described.

### Selection of genomic regions for creating targeted mutations

For creating targeted insertion mutations, several genomic regions spanning both intergenic and intragenic regions were selected. The genomic targets within protein coding sequences included genes Ech_0126 (a hypothetical protein gene), Ech_1136 (p28-Omp 14 gene) and Ech_1143 (p28-Omp 19 gene). The intergenic regions included the DNA segments spanning the genes Ech_0039 and Ech_0040, Ech_0111 and Ech_0112, and Ech_0251 and Ech_0252. The hypothetical protein genes were selected because our RNA analysis (not shown) suggested that the bacterium did not transcribe from these genomic regions when propagated in DH82 macrophages. Ech_1136 is a major transcriptionally active gene encoding the p28-Omp 14 protein when the pathogen is grown in tick cells, whereas Ech_1143 gene is highly active in producing the p28-Omp 19 protein during its replication in vertebrate macrophages [15]–[18]. The underlying hypothesis for selecting these two differentially expressed genes is that disruption of a gene expressed exclusively in tick cells should permit normal growth of the organism in macrophages. Similarly, insertion mutations of genes expressed in macrophage environment should allow the pathogen to grow normally in tick cells.

### Insertion mutations by homologous recombination

Two types of recombinant DNA segments were prepared targeting the gene Ech_0126; in the first version (Rec I), a segment was prepared to introduce an antibiotic resistance gene to disrupt the gene. The recombination event results in the increase of a genome size by 0.93 kb. As the increase in genome size may cause polar effects influencing expression of genes surrounding the insertion site, a second type of recombinant segment was created to minimize the genome size increase in mutants. In this strategy (Rec II), the length of the Ech_0126 segment was reduced to approximately equal the length of the antibiotic resistance cassette introduced. The homologous recombination schemes to achieve this are presented as a cartoon in supplemental Figure 1 (Figure S1). Independent of the recombination methods used, mutants were identified which persisted up to six days in culture and the presence of antibiotic had no impact on the mutants recovery; the mutants were assessed by Southern blot analysis of the PCR products generated with primers specific to the *E. chaffeensis* genomic region upstream of the insertion site and the inserted CAT gene sequence (Figure S2). Insertion junctions were verified by sequencing of the PCR products (data not shown). Similar experiments were performed with gentamicin resistance cassettes and the mutants were detected similarly for six days.

**Figure 1:**
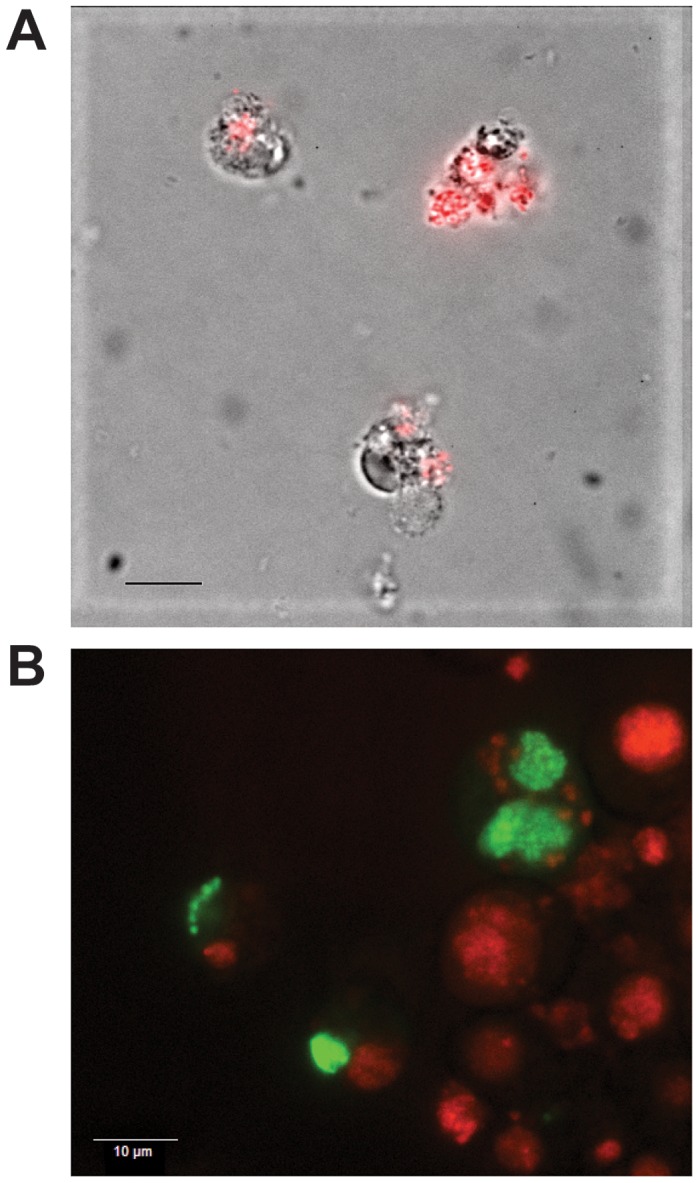
Expression of GFPuv and mCherry in transformed *E. chaffeensis*. Himar1 transposon mutants of *E. chaffeensis* were generated following electroporation of the constructs into the cell-free organisms recovered from infected ISE6 tick cells and re-cultured in macrophage and tick cells. A drop of culture suspension under a cover slip was imaged under uv-illumination using an Olympus BX61 spinning disk confocal microscope and a Qfire digital camera. Panels A and B represent *E. chaffeensis* Arkansas isolate expressing mCherry and GFPuv, respectively. Bars = 10 µm.

We reasoned that the lack of persistently growing mutants in culture may have resulted because the genomic region selected for the homologous recombination may be necessary for the bacterium's continued growth. Alternatively, the antibiotic resistance cassettes containing *E. coli* codon usage may not be optimal for protein expression in *E. chaffeensis*. We modified the codon usage for the CAT and gentamicin resistance gene cassettes to optimize their expression in *E. chaffeensis* and the mutational experiments were repeated. The targeted insertion mutants were detected similar to non-codon optimized constructs, but as in previous experiments the mutants survived only for up to eight days (not shown).

### Insertion mutations by employing TargeTron mutations

We expanded the targeted mutational experiments to six genomic sites by utilizing a mobile group II intron-based mutagenesis method (the TargeTron method) [25]–[28]. We opted to utilize this method as many studies in recent years reported its application for creating targeted mutations in several Gram-negative and Gram-positive bacteria [29]–[31]. The mobile group II intron was modified for insertions at six genomic locations of *E. chaffeensis*, including into the coding regions of Ech_0126, Ech_1136, and Ech_1143 and into intergenic regions of the genes Ech_0039 and Ech_0040, Ech_0111 and Ech_0112, and Ech_0251 and Ech_0252. We inserted the pathogen's *tuf* promoter to promote expression of modified group II intron RNA and intron encoded protein in *E. chaffeensis*. The CAT gene cassette was also part of the modified mobile group II introns to confer resistance against chloramphenicol. Multiple assessments of insertions in all of the target regions led to the consistent identification of mutants when the mobile group II intron was targeted to the genes Ech_0126, Ech_1143 and to the non-coding region between the genes Ech_0111 and Ech_0112, but not in other three locations. Similar to the observations for the homologous recombination at Ech_0126 gene, the mutants persisted only for up to 8 days in culture. The presence of insertions was confirmed by Southern blot analysis of the PCR products generated with primers specific to insertion segment and to the surrounding genomic regions (Figure S3).

### Random insertion mutations created with the Himar1 transposase

Recent studies have demonstrated that Himar1 transposon mutagenesis can be reliably achieved in a rickettsial organism closely related to *E. chaffeensis*, i.e., *A. phagocytophilum* [32]. In creating random mutations in *E. chaffeensis*, we utilized Himar1 transposon constructs similar to the ones used for mutagenesis of *A. phagocytophilum* [32]; the constructs prepared for this study represent single plasmids containing the Himar1 transposase gene and a transposon segment comprising of genes encoding mCherry or green fluorescent protein (GFPuv) co-expressed with the *aad* gene to confer resistance to spectinomycin and streptomycin flanked by inverted repeats recognized by the transposase. Three independent electroporation experiments were performed (once with the mCherry plasmid and twice with the GFPuv plasmid) using ISE6 tick cell-derived *E. chaffeensis* organisms; the organisms were then propagated in both ISE6 cells and DH82 cells. Antibiotic resistant organisms that persisted in culture for several months were identified using dual selection with spectinomycin and streptomycin (100 µg/ml each). The cultures also expressed mCherry or GFPuv (Figure 1).

**Figure 2:**
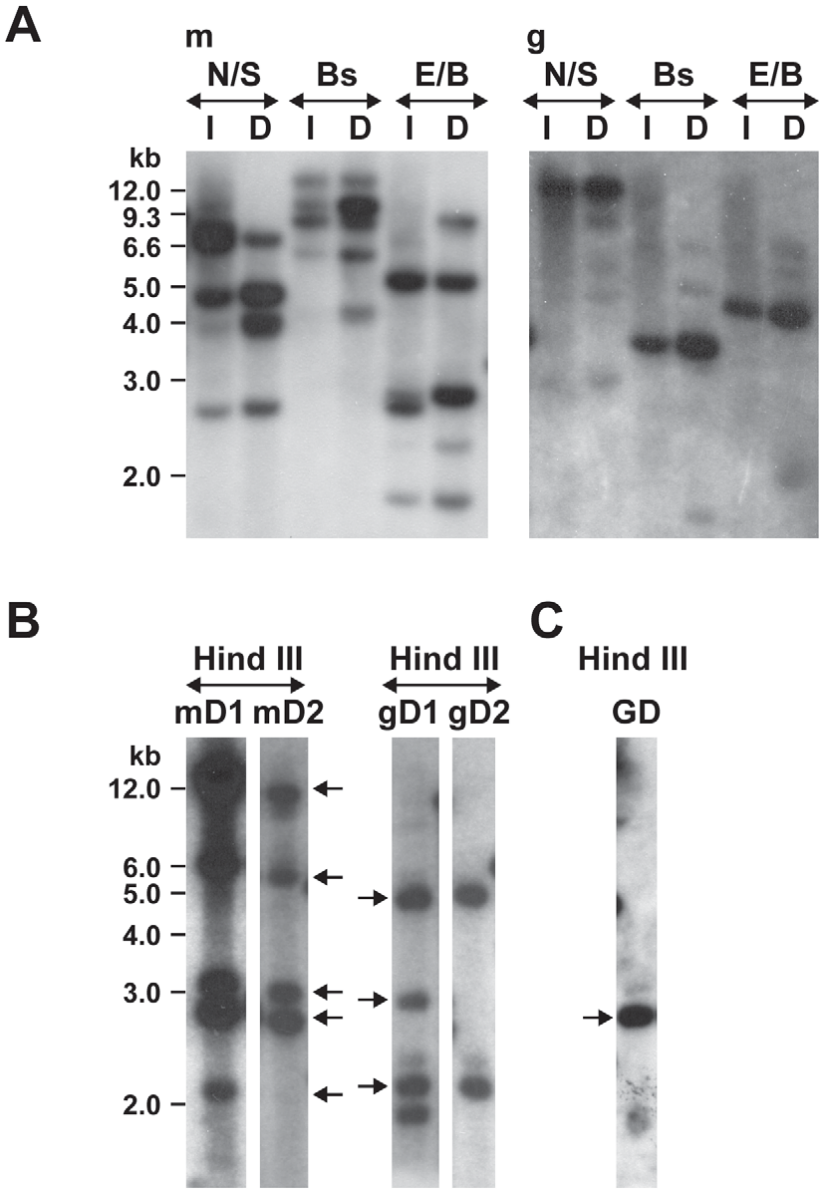
Southern blot analysis of Himar1 transformants of *E. chaffeensis*. Genomic DNA from Himar1 transposon *E. chaffeensis* mutants was assessed by DNA blot analysis using a spectinomycin resistance gene (*aad*) probe following digestions with different restriction enzymes (N, Nde I; S, Spe I; Bs, Bsrg I; E, EcoR V; B, Bgl II and Hind III). A) *E. chaffeensis* genomic DNA was recovered from the mutant organisms of mCherry plasmid transformed (m); GFPuv plasmid transformed (1^st^ experiment) (g) grown in ISE6 tick cells (I) or DH82 macrophages (D). B) Genomic DNAs from the mCherry and GFPuv (1^st^ experiment) mutants propagated in DH82 cultures was also assessed at two different randomly selected harvest times (separated about 1 month apart) to evaluate the stability of the transformants over time. The lanes mD1 and mD2 represent two different days when mCherry mutants were harvested, and gD1 and gD2 represent different harvest dates for GFPuv mutants. C) Second GFPuv plasmid transformed mutants grown in DH82 culture and assessed by DNA blot analysis (GD). Genomic locations for the DNA fragments indicated by the arrows were established by sequence analysis. Values shown on the left side of panels represent DNA size markers.

The transposon insertions in the mutants were verified by Southern blot analysis performed with an *aad* gene probe (Figure 2). Several restriction enzymes lacking recognition sites in the region that bound the DNA probe were used in the analysis. Several insertion sites were observed in the mCherry mutant DNA, as judged from the Southern blot signals (both weak and strong signals). Similarly, multiple insertions were observed in the first GFPuv mutant DNA. In the second transformation experiment with the GFPuv plasmid, we recognized only one strong insertion segment. The intensity and the presence or absence of the hybridized DNA fragments in the mCherry and in the 1^st^ GFPuv plasmid transformants were variable in DNA isolated from the organisms recovered from infected macrophages or tick cells (Figure 2, panel A). Similarly, hybridized fragments were variable in cultures when harvested at different times following electroporation (data were presented for the cultures grown in macrophage cell line) (Figure 2, panel B).

**Figure 3:**
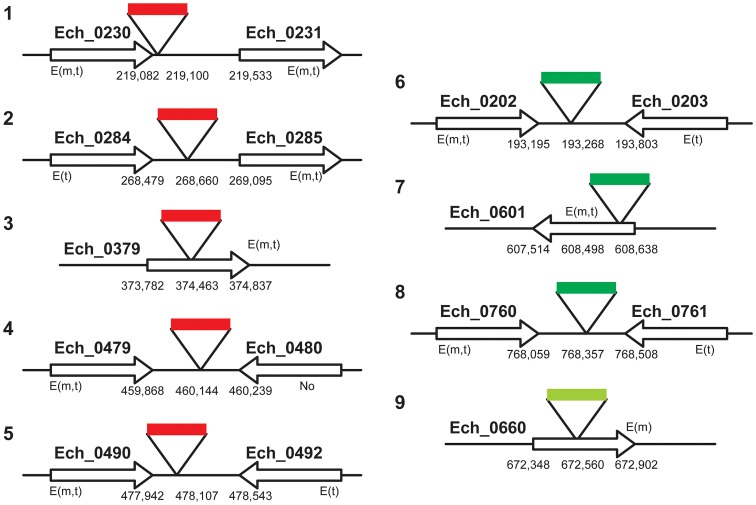
A cartoon illustration of the *E. chaffeensis* genomic locations mapped for the transposon mutants. *E. chaffeensis* genomic DNA from three independent transformations with mCherry (one transformation) and GFPuv (two transformations) Himar1 transposon plasmids was used to determine the integration locations by inverse PCRs and ST-PCRs followed by DNA sequence analysis. Genomic locations of the insertion sites and the genes at or near the insertions, as per the whole genome data (GenBank # CP000236.1), were presented. The gene expression data assessed by RT-PCR were also included in the figure (E, expressed gene; m, in macrophage culture; t, in tick cell culture; No, gene not expressed). The insertions in mCherry transformants are shown as solid red bars, and insertions in GFPuv transformants are depicted as solid green bars (dark green bar, the first GFPuv transformants; light green bar, the second GFPuv transformant).

**Figure 4:**
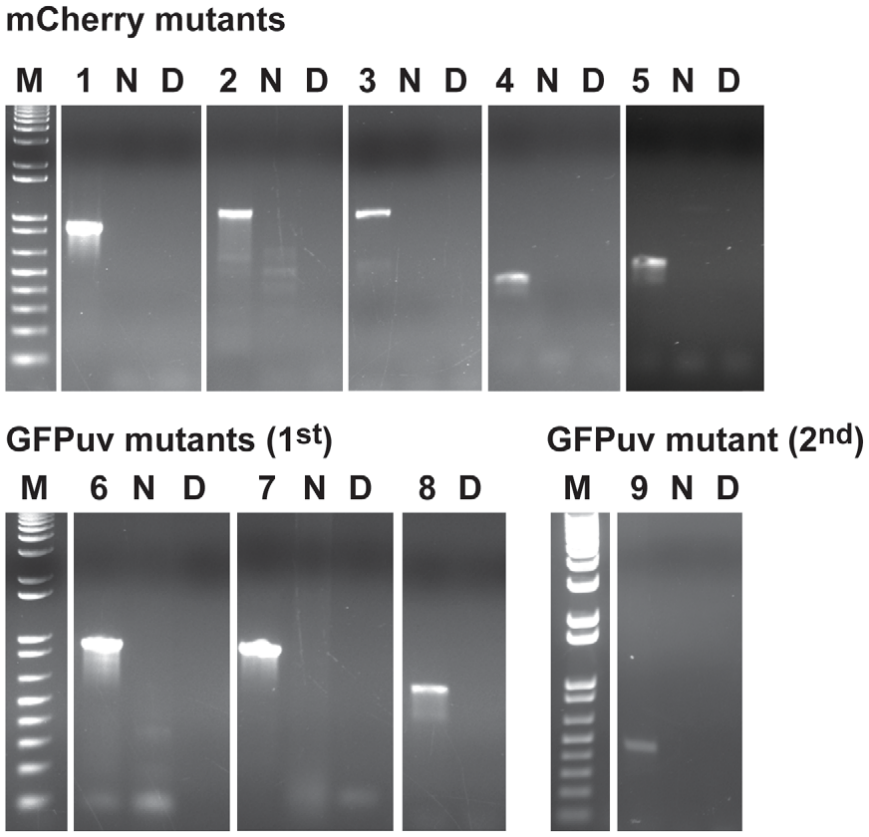
Validation of transposon insertions in the *E. chaffeensis* genome. The insertion sites in the *E. chaffeensis* genome were verified by PCR with primers designed to bind to the genomic region upstream of the insertion sites (forward primer) and to the inserted DNA (spectinomycin resistance gene) (reverse primer). Product sizes for all 9 insertions are different and the predicted size amplicons were observed only in PCRs containing the mutant genomic DNAs as the templates. Lanes 1–9 represent different insertion targets (as listed in Figure 3) amplified from the mutant genomic DNAs of mCherry mutants (lanes 1–5), and GFPuv culture DNAs from the first (lanes 6–8) and second (lane 9) transformation experiments; N, no template control; D, wild type *E. chaffeensis* DNA used as the template; M, 1 kb+ DNA molecular weight marker.

To map the genomic locations of the transposon insertions, inverse PCRs and ST-PCRs (semi-random, two-step PCR) followed by sequence analysis of the PCR products were performed. We mapped five genomic locations of the mCherry transposon inserts and four genomic locations from the GFPuv insets (three from the 1^st^ and one from the 2^nd^ GFPuv transformants) (Figure 3). Six of the 9 transposon insertions were present within the non-coding regions of the genome; one of the insertions is close to the termination codon of the gene Ech_0230 (just 18 base pairs 3′ to the termination codon). The remaining three insertions were present within the coding regions of three hypothetical protein encoding genes Ech_0379, and Ech_0601, and Ech_0660. The insertions at all 9 genomic sites were confirmed by PCR and sequence analysis with primers designed to bind to the regions targeting the inserted DNA and to the surrounding genomic locations (Figure 4).

**Figure 5:**
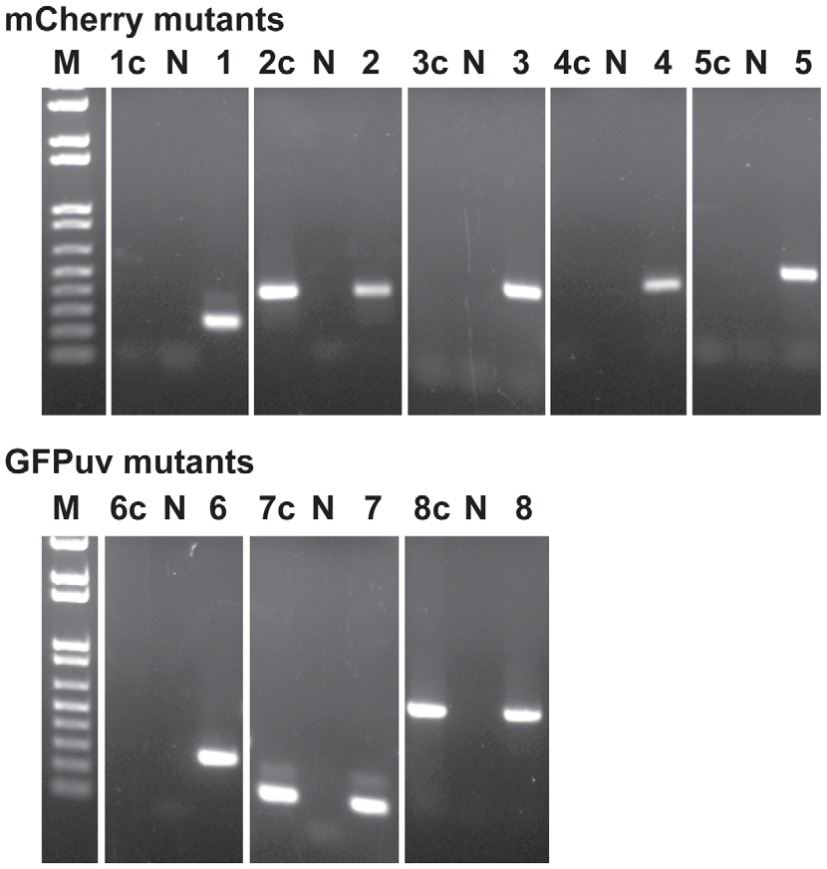
Clonal isolation of the *E. chaffeensis* mCherry and GFP mutants. Host cell-free organisms recovered from nearly 90% infected culture flask were used to make 10-fold serial dilutions and cultured in DH82 cultures. The culture flasks resulting in *E. chaffeensis* growth from the highest dilution were used to isolate genomic DNA and for assessing the Himar1 insertions by performing insertion-specific PCRs. Lanes 1–8, genomic DNA from mixed populations of mutants prior to cloning by serial dilution was used as the template for PCRs targeting to the 8 Himar genomic insertion sites (as listed in Figure 3); Lanes 1c–8c, as in lanes 1–8, but using DNAs recovered after serial dilution cloning of the organisms; Lanes with the letter N refer to no template DNA controls for each of the PCRs and lane M contained the 1 kb+ DNA molecular weight marker.

Himar1 transposon insertions occur mostly once or twice per genome [36]–[38]. In this study, we observed multiple insertions in the mCherry transposon mutants and similarly for the first experiment performed with the GFPuv construct. We reasoned that the mutants represented heterogeneous mixture of organisms; this is consistent with varied signals in the Southern blots for the mutants isolated at different times in culture and for the observed differences in mutants recovered from macrophage and tick cell cultures (Figure 2). To test the hypothesis that the mutants represent a heterogeneous mixture, we performed serial dilution culture experiments with 10 fold dilutions of cell-free organisms mixed into uninfected macrophage cultures, and the infection in higher dilutions (an estimated number of bacteria in the range of 0.1 to 10 per well) was monitored for growth. We used Southern blotting (not shown) and PCR to target all 8 genomic insertions in cultures that tested positive for bacterial growth (Figure 5). The PCR products were observed for the clonal population only for the insertion downstream to the Ech_0284 (lane 2c in Figure 5) from the mCherry mutant pool following the dilution cloning experiment. Similar efforts in recovering clonal populations from GFPuv mutant cultures resulted in the elimination of one of the three mutants from the pool (mutant downstream to the Ech_0202) (Lane 6c in Figure 5)

### Mutations within the protein coding regions resulted in loss of gene activities

**Figure 6:**
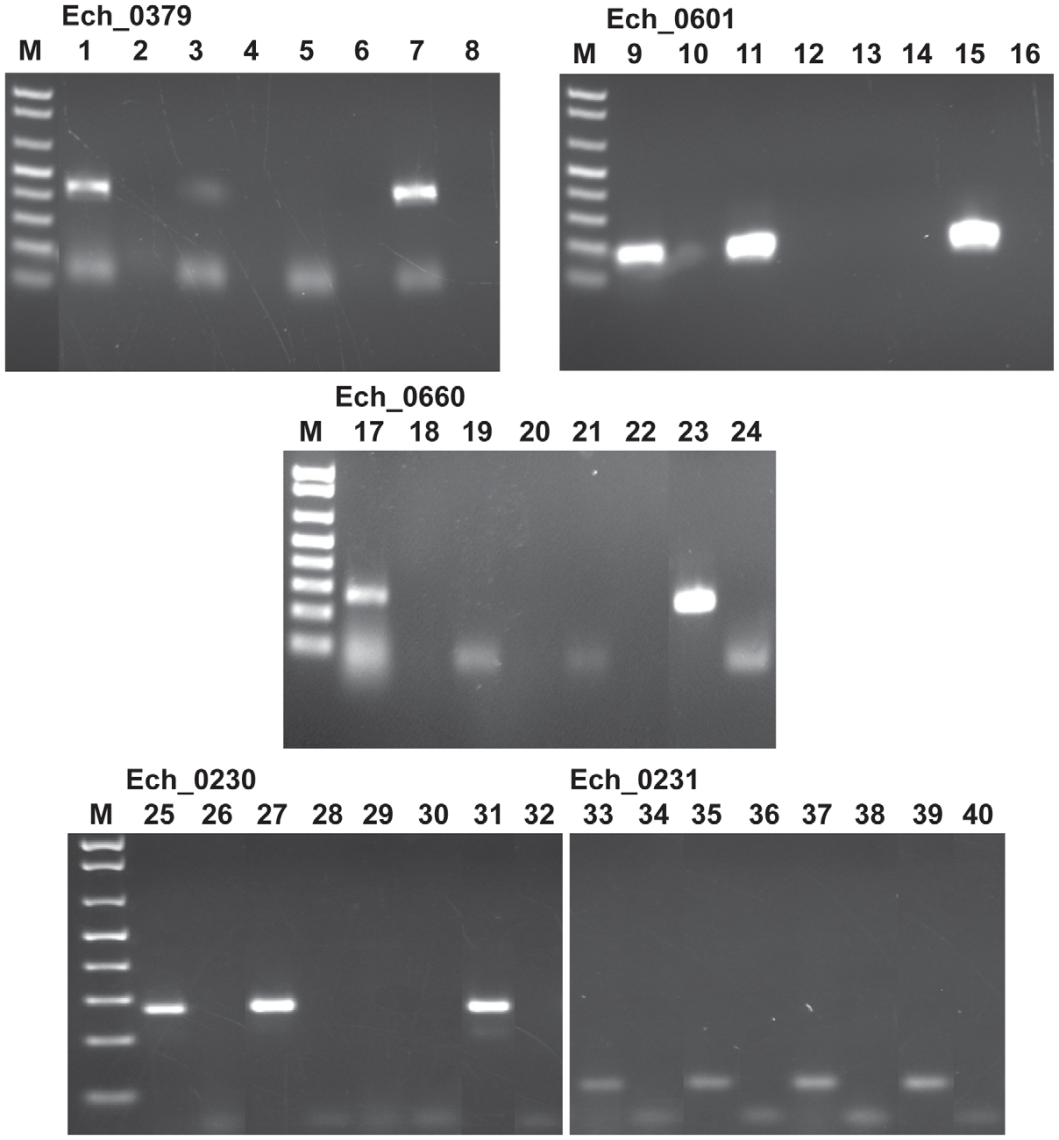
Transcriptional analysis of the transposon mutants with insertions within the protein coding regions of Ech_0379, Ech_0601, Ech_0660 and Ech_0230 genes. Total RNA isolated from wild type *E. chaffeensis* grown in DH82 culture (lanes 1, 2, 9, 10, 17,18, 25, and 26) and AAE2 tick cell culture (lanes 3, 4, 11, 12, 19, 20, 27, and 28) and the mCherry mutant culture derived (lanes 5,6, 29, and 30) and GFPuv first transformation (lanes 13 and 14) and second transformation (lanes 21 and 22) cultures grown in DH82 cultures was assessed by RT-PCR for evaluating the gene expression. PCR products resolved in lanes 1–8 included primers targeted to the Ech_0379 and lanes 9–16 contained primer set for the gene Ech_0601, lanes 17–24 contained primers targeted to Ech_0660, and lanes 25–32 had primers targeted to Ech_0230. (Lanes 2, 4, 6, 10, 12, 14, 18, 20, 22, 26, and 28 did not include reverse transcriptase; lanes 7, 15, 23, and 31 served as the positive controls as they included the wild type genomic DNA; and lanes 8, 16, 24, and 32 contained no template and served as negative controls.).

Three of the 9 transposon mutants included insertions into the protein coding regions of three different hypothetical protein genes; Ech_0379, Ech_0601 and Ech_0660. To evaluate if the insertions resulted in the loss of gene activity from these genes, mRNA recovered from the wild-type and mutant organisms grown in culture was assessed by RT-PCR. All three genes are transcriptionally active in wild-type bacteria replicating in macrophages and two of the three genes are also active during growth in AAE2 tick cells (Ech_0379 and Ech_0601) (Figure 6). Transcript levels for Ech_0379 were, however, low in the tick cell-derived bacteria, whereas Ech_0601 expression levels were similar in both macrophages and tick cells. Transposon insertions into the coding sequences of all three genes resulted in complete loss of gene expression (Figure 6) without impacting growth in macrophage or tick cell cultures *in vitro*. As the insertion mutation near Ech_0230 is only 18 base pairs downstream from its coding sequence, we also assessed the impact of this mutation on the gene expression. This gene is expressed in wild-type organism grown in both the macrophage and tick cell cultures. The mutation, however, resulted in the loss of gene expression (Figure 6).

### Impact of mutations on the pathogen's growth in vertebrate and tick hosts

We investigated whether one or more mutations into the coding or non-coding regions affected the pathogen's growth and persistence in a mammalian host or its acquisition by tick. A laboratory reared, white-tailed deer was inoculated intravenously with a mixed (i.e., not a clonal population) mCherry transformant culture containing all five different insertion mutants. The presence of mutants in the inoculum was confirmed by Southern blot and insertion-specific PCRs (not shown). Deer blood was monitored for infection by culture recovery of the organisms and PCR analysis targeting to all five insertion sites and for the presence of spectinomycin resistance gene for up to 63 days. With the exception of a few post-infection dates, the animal tested positive for the culture recoverable pathogen and insertion-specific, spectinomycin resistance gene (*aad*) (Table 1) throughout the study period, suggesting persistence of the mutant organisms. The pathogens recovered from deer also expressed functional mCherry protein similar to the mutants in culture prior to infecting the animals (data not shown). Evaluation of deer blood-derived total genomic DNA by insertion-specific PCRs targeting to all five genomic locations originally identified in the mCherry transformants resulted in the amplification of segments only from three intergenic locations (Table 1). Amplicons diagnostic for the insertion within the coding region of the gene Ech_0379 and one insertion into the non-coding region located 18 base pairs downstream from the Ech_0230 coding sequence were negative in all blood samples analyzed. Furthermore, the culture recovered organisms from blood samples evaluated by insertion-specific PCRs confirmed the presence of mutations only at the same three non-coding regions (not shown). To confirm these results, we repeated the deer-infection experiment using mCherry transformants in two additional animals, and followed the infection for 49 days. As in the first experiment, mutants persisted in deer included only the same three intergenic insertions, whereas transformants with mutations into the coding region of Ech_0379 and the non-coding region mutation at the 3′ end of the Ech_0230 termination codon were not detected (Table 2).

**Table 1:** Verification of the *E. chaffeensis* infection status by culture and for the transposon insertion sites in mCherry mutant infected deer blood#.

	Days post infection*											
	0	6	8	10	14	18	21	28	39	52	59	63
Culture	−	+	+	+	+	+	+	+	+	+	+	+
*Aad*	−	+	+	+	+	+	+	+	+	−	−	+
1**	−	−	−	−	−	−	−	−	−	−	−	−
2**	−	−	−	−	−	−	+	−	−	−	−	−
3**	−	−	−	−	−	−	−	−	−	−	−	−
4**	−	+	−	+	+	+	+	+	−	+	+	−
5**	−	−	−	−	+	+	+	+	−	−	−	−

# First infection experiment.

* Blood-derived buffy coats or DNAs from the first infection experiment were assessed for the presence of mutants by culture recovery and nested PCR, respectively. Culture positives refer the presence of organisms as inclusions in the DH82 cells as determined by cytospin and polychromatic stained slides. Blood recovered on days 5, 32, 35, 42, 46, 49 post-infection were also assessed and tested negative for the viable pathogen or predicted amplicons. The − and + signs refer to samples tested negative or positive.

** Genomic insertion sites as defined in Figure 3.

**Table 2:** Nested PCR verification of the *E. chaffeensis* infection status and for the transposon insertion sites assessed in deer blood DNA#.

	Days post infection										
Animal 1	0	3	7	10	13	17	20	23	30	37	49
*Aad*	−	+	−	−	+	+	−	+	+	+	−
1**	−	−	−	−	−	−	−	−	−	−	−
2**	−	+	−	−	−	+	−	−	−	−	−
3**	−	−	−	−	−	−	−	−	−	−	−
4**	−	−	+	−	−	−	−	+	−	+	+
5**	−	−	−	−	+	+	−	+	+	−	−
Animal 2											
*Aad*	−	−	−	−	+	+	−	+	+	−	+
1**	−	−	−	−	−	−	−	−	−	−	−
2**	−	−	−	−	−	−	−	+	+	−	+
3**	−	−	−	−	−	−	−	−	−	−	−
4**	−	−	−	−	−	+	+	−	+	+	+

# Second infection experiment;

** Genomic insertion sites as defined in Figure 3.

Nymphal *A. americanum* ticks were allowed to feed on a deer starting on day five post infection; fully engorged ticks that dropped off the animal were allowed to molt. Ten randomly collected molted adult ticks of both sexes were tested for *E. chaffeensis* transformant DNA by nested PCR targeting the spectinomycin resistance gene. Seven ticks that tested positive for the mutants were further assessed for the presence of mutations at all five genomic insertion sites (Table 3). The tick DNAs tested PCR positive for insertion mutations in two intergenic locations, and tested negative for the insertion within the coding region of the gene Ech_0379 and the non-coding insertion near Ech_0230 gene coding sequence, as observed from the PCR results for deer blood. The ticks were also negative for one other insertion located in the non-coding region between the genes Ech_0284 and Ech_0285.

**Table 3:** Nested PCR verification of the *E. chaffeensis* infection status and for the transposon insertion sites in ticks* fed on infected deer.

Target	Tick2	Tick4	Tick5	Tick6	Tick7	Tick8	Tick 9
*Aad*	+	+	+	+	−	+	−
1**	−	−	−	−	−	−	−
2**	−	−	−	−	−	−	−
3**	−	−	−	−	−	−	−
4**	+	+	+	+	−	+	+
5**	−	−	+	+	+	+	+

* Ten ticks were assessed and three of which tested negative for the pathogen.

** Genomic insertion sites as defined in Figure 3.

The mutant pool infection experiments described above are similar to studies reported in the literature in identifying virulence associated genes by negative selection of several bacterial pathogens [39]–[41]; it will be a valuable tool for identifying additional genes essential for the *E. chaffeensis* infection *in vivo*. To further validate that the negative selection approach aids in identifying essential genes of *E. chaffeensis*, we used two clonal populations of mutants in another infection study in deer. One of these mutants had insertion within the coding region of the gene Ech_0660 and the second one has a mutation in the intergenic region located between the genes Ech_0284 and Ech_0285. This clonal pair was selected because the Ech_0660 mutant was identified as having single insertion in the genome (Figure 2) resulting in the loss of gene expression (Figure 6) and the mutant in the intergenic region between Ech_0284 and Ech_0285 is one of the mutants that grew continuously in infected deer when inoculated as part of the pool of mutants (described above). Blood samples collected from the infected animals for up to 20 days were assessed to monitor the mutants in circulation by performing nested PCR analysis (Table 4). The Ech_0660 infected animals tested negative for blood samples assessed for all post-infection days, whereas the infection with the intergenic mutant clone persisted in deer, as evidenced by the PCR positives on several post-infection days.

**Table 4:** Nested PCR verification of the *E. chaffeensis* infection with clonal population mutants in deer blood.

Days post infection								
*Mutant #	Animal #	0	3	6	9	13	16	20
2	1	−	−	+	+	−	+	+
2	2	−	−	−	+	+	+	−
9	3	−	−	−	−	−	−	−
9	4	−	−	−	−	−	−	−
9	5	−	−	−	−	−	−	−

* Clonal population mutants 2 (mutation in the noncoding region between the genes Ech_0284 and Ech_0285) and 9 (mutation in the gene Ech_0660) as shown in Figure 3.

## Discussion

*Ehrlichia* species cause important diseases in people and animals, and understanding the pathogenesis and host-specific differences in gene expression of these pathogens are important goals. However, a major hurdle in advancing research in *Ehrlichia* species has been the lack of genetic tools, specifically, reliable methods for mutagenesis. This may largely be due to the fact that these organisms can survive only for a very short period of time outside a host cell [20]. Moreover, these phagocytotropic organisms do not appear to harbor plasmids [42], and may not be amenable to plasmid transformation. Although preliminary evidence resulting from attempts to mutagenize *E. chaffeensis* have been discussed at scientific meetings [43], [44], stable mutants have not been realized until now. In this study, we took a methodical approach in demonstrating the feasibility of creating both targeted and random mutations in *E. chaffeensis*. Firstly, we evaluated various experimental conditions to identify protocols that work the best in transforming the organism with a plasmid (results not discussed). Secondly, we assessed various antibiotics in support of identifying drugs that are useful in monitoring the mutants. We then tested the feasibility of creating targeted mutations by homologous recombination at a site which appeared to be transcriptionally silent. We subsequently expanded the targeted mutagenesis to six genomic locations using the modified mobile group II intron method, as recent research had demonstrated that it is an efficient method for creating targeted mutations in several Gram positive and Gram negative bacteria [28]–[31]. The method aided in the consistent identification of mutations in three targets assessed, including one located within a differentially expressed gene (Ech_1143) that encodes an outer membrane protein, p28-Omp 19. The mutants, however, survived in culture only for a short period of up to 8 days. We then evaluated transposon-based random mutagenesis and demonstrated that stable *E. chaffeensis* mutants could be generated in this way. Random mutagenesis resulted in multiple mutations at several genomic locations. The random mutants included within the coding regions of two differentially expressed genes (Ech_0379 and Ech_0660) encoding for two different hypothetical proteins and one in the coding region of a constitutively expressed hypothetical protein gene (Ech_0601). Together, the experiments described in this study demonstrate that it is possible to create mutations within *E. chaffeensis* genes in both constitutively and differentially expressed genes, as well as in intergenic regions.

The choice of mutagenesis methods does not seem to have any impact in creating mutations at a specific region of the genome. For example, we used three independent methods in creating targeted insertion mutations into the Ech_0126 gene (two different approaches of homologous recombination and a TargeTron method). The use of all three methods aided in the identification of disruption mutations within this gene. It is not clear why the targeted mutations did not survive longer in culture; several possibilities should be considered: 1) it is possible that the genomic regions selected for insertion mutations may represent regions that are necessary for the organism's normal persistent growth in culture. 2) Alternatively, the *E. chaffeensis* promoter (*rpsl*) utilized in driving the expression from CAT gene may not be optimal in *E. chaffeensis* conferring resistance against the antibiotic concentration used. Although the *rpsl* promoter worked well in developing resistance in *E. coli* in driving the CAT gene expression (not shown), its expression in *E. chaffeensis* may be an issue due to duplication of the *rpsl* promoter sequence in the genome. We are now investigating the use of heterologous promoters in targeted mutation experiments, such as the *A. marginale tr* promoter, as we already demonstrated its utility in the current study in the transposon mutagenesis in *E. chaffeensis*.

Our efforts to create targeted mutations resulted in the establishment of mutants only in three out of six genomic targets evaluated. We reasoned that the targets selected for the three regions where mutants could not be detected might represent essential regions of the genome. One in particular, the Ech_1136 gene encodes for a protein (p28-Omp14) that is differentially expressed with significant expression being observed in tick cells *in vitro* and *in vivo* [15]–[17], [45], [46]. The p28-Omp 14 is also a highly immunogenic protein; we reasoned that this gene product is an important component and its expression is critical for the organism's survival in a tick cell environment, but may be less critical for its growth in macrophages. The mutational analysis, however, suggested that the insertion mutation into this gene results in the complete inhibition of *E. chaffeensis* growth; this conclusion may infer that this gene product is necessary for the organism's growth in macrophages under *in vitro* conditions. This hypothesis is consistent with our recent studies suggesting that the protein from this gene is also expressed in macrophages [15]. Interestingly, targeted mutation in the predominantly macrophage-specific outer membrane protein gene of p28-Omp 19 (Ech_1143), a paralog of the p28-Omp 14 gene, appears to have no impact on the growth of the organism in macrophage cultures.

Southern blot analysis of DNA recovered from transposon mutants harvested at different times during their growth in macrophage or tick cell cultures suggested that the mutants are a heterogeneous population. The Himar1 transposase mutations typically occur only at one or two locations in a genome [36]–[38]. To verify that the Himar transposon mutations in *E. chaffeensis* are similarly occurring at fewer genomic locations, we attempted to clone the mutants by performing a serial dilution cloning experiment. Establishing clonal populations from mutant pools is challenging and is a time consuming task for intracellular pathogens, such as *E. chaffeensis.* However, we were able to identify a single insertion containing culture from the mCherry mutant population and could eliminate one mutant from the GFPuv mutant pool.

We mapped 9 random insertion mutation sites in the *E. chaffeensis* genome; six were located within non-coding regions and three were present within coding regions of three different transcriptionally active genes, and the mutations within the coding regions inhibited the mRNA production. Two of the active genes disrupted by the Himar1 insertion were differentially expressed genes [expressed highly (Ech_0379) or exclusively (Ech_0660) in macrophage cultures]. The complete inactivation of the genes caused by these mutations did not affect the growth of the organisms in either macrophages or tick cells. The nine genomic locations are the first to be identified as non-essential for *E. chaffeensis* growth *in vitro* in both vertebrate and tick cells; however, not all mutants grew in white-tailed deer (the natural reservoir host for the pathogen [47]), and not all were acquired by its transmitting vector tick, *A. americanum* [47], [48]. In particular, the transposon insertion into the coding region of the hypothetical protein encoding genes, Ech_0379 and Ech_0660, and into the non-coding region near the 3′ end of Ech_0230, prevented growth of the mutant organisms in deer. We were puzzled with the observation that the insertion mutation in a non-coding region also limited the growth of the organism in deer. One possibility is that insertions at certain non-coding regions of a genome may cause a polar effect influencing gene expression from surrounding genes leading to abolished growth *in vivo*, similar to the observations noted in the literature for other organisms [49]. Our RNA analysis, however, revealed that the mutation at the 3′ end of the ECH_0230 caused the inactivation of gene expression from this gene. The 3′ end mutation may have altered the transcript stability of Ech_0230, possibly because it may be located within the DNA segment representing the transcriptional unit.

**Figure 7:**
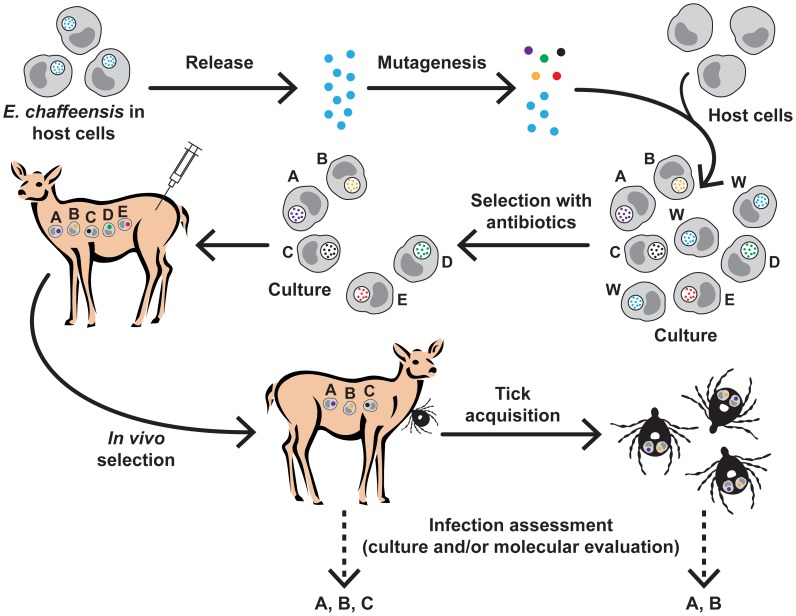
Schematic representation of the preparation of *E. chaffeensis* transposon mutants and *in vivo* screening to identify genes important for the pathogen's growth in deer and tick hosts. The protocol involves recovering *in vitro* culture-derived *E. chaffeensis* organisms, subjecting them to transposon mutagenesis, reinfecting to naïve host cells, selecting the mutants in culture resisting to antibiotic clearance, infecting a natural host (white-tailed deer), acquisition feding of ticks on the deer and finally assessing the mutants survived in deer and ticks.

We performed the infections in deer with pool of mutants in three separate animals using two independently cultured batches of organisms and the analysis also included 31 blood samples collected at various post-infection days (10 or 11 harvest points from each animal). All five mutants were represented in the culture pool used for infections. Independent of a blood sample analyzed, the only mutants consistently not detected in deer blood were those with the loss of gene expression (in genes Ech_0230 and Ech_0379). The *in vivo* survived mutants in deer, therefore, represent only those for which the transcriptional activity was not negatively affected in genes near the insertion mutations. More importantly, mutations causing the loss of gene expression correlated very well with their failed growth in deer or their non-acquisition by ticks during blood feeding. Together, these data suggest that the elimination of some mutants *in vivo* from a pool of mutants is the result of the *E. chaffeensis* growth inhibition because of the loss of gene function probably impacting the survival of the pathogen *in vivo*. The protocols outlined in this study, beginning from creating mutations and assessing their ability to grow or not to grow in a host (Figure 7), will serve as a useful tool in screening the genome of *E. chaffeensis* to identify additional genes contributing to the pathogenesis. The *in vivo* infection assessment with pools of mutants to identify virulence associated genes in bacterial pathogens is well-documented in the literature [39]–[41]. Our study, however, is the first to apply similar strategy for an obligate intra cellular rickettsial pathogen and it is likely valuable in initiating similar studies in other related pathogens. The method may also be applicable for other obligate intracellular pathogens, such as Chlamydia for which genetic manipulation methods are still at infancy [50].

Establishing clonal populations from mutant pools is a challenging task for intracellular pathogens, such as *E. chaffeensis*. We, however, were able to prepare two clonal populations from the mutants described in this study; one having mutation causing the loss of gene expression (Ech_0660) and the second one having a mutation into a noncoding region (between the genes Ech_0284 and Ech_0285) with no impact of gene expression in genes 5′ and 3′ to the insertion mutation (data not shown). Infections with the two clones in deer further supported the conclusions drawn from the infection experiments using pool of mutants. The Ech_0660 mutant causing the gene expression inactivation also failed to grow in deer, while the intergenic mutant persisted well. The lack of growth of the subset of mutants from a pool of mutants in deer, therefore, is not a random event because the only mutants that failed to grow in deer are those impacting the gene expression of the organism. Ticks also did not acquire *E. chaffeensis* mutants containing gene function disruptions. One other mutant with insertion into the non-coding region between the genes Ech_0284 and Ech_0285 also did not grow in ticks. Failure of ticks to acquire the gene disruption mutants is likely due to their inability to productively infect deer during the time of tick feeding. The third mutant (mutant # 2) that infected deer but was not acquired by ticks, may represent an insertion mutation at a genomic region required for *E. chaffeensis* growth in ticks. The alternate possibility is that the specific mutant organisms were not in circulation in deer during the period of tick feeding.

**Figure 8:**
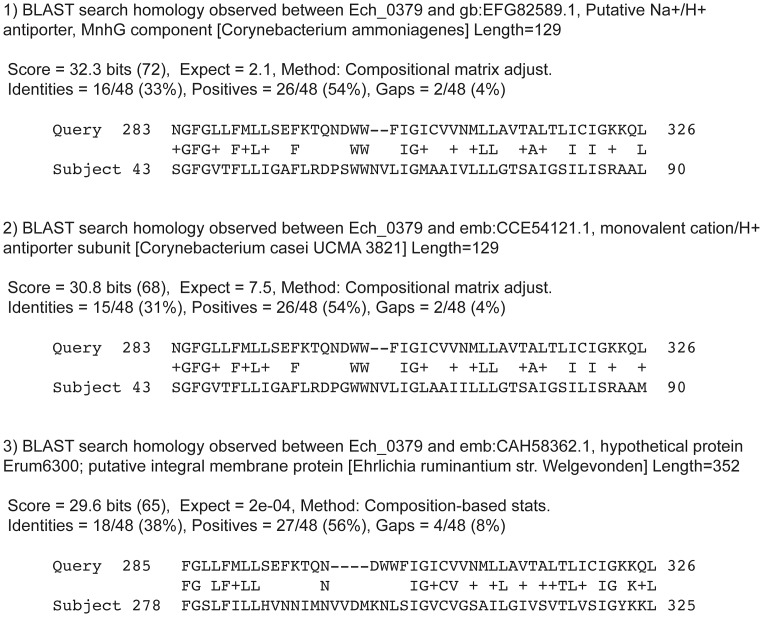
Basic Local Alignment Search Tool (BLAST) homology search analysis of Ech_0379. The translated protein coding sequence of Ech_0379 was subjected to BLAST analysis by searching against protein databases available through the National Center for Biotechnology Information (NCBI) web site. The homologies having significant sequence similarities with identities above 30% and positives above 50% to a sequence motif in the Ech_0379 were identified and presented. Three significant hits were presented in the Figure.

Specific functions of the disrupted genomic regions remain to be determined. The gene Ech_0230 encodes for a putative membrane protein and Ech_0379 and Ech_0660 are identified as genes encoding for hypothetical proteins [38] (GenBank # CP000236.1). The gene product for the Ech_0660 contains a putative conserved domain having significant homology to phage proteins involved in the phage capsid assembly (observed in the BLAST search analysis), suggesting that it may represent a membrane expressed protein of *E. chaffeensis.* Protein database homology search on the translated coding sequence of Ech_0379 revealed a significant homology for a 46 amino acid long N-terminus domain with a membrane-bound, monovalent cation (Na^+^)/H^+^ antiporter protein subunit of two *Corynebacterium* species; *C. ammoniagenes* and *C. casei* (Figure 8). Ech_0379 gene homologs are also conserved in other *Ehrlichia* species, e.g., in *E. ruminantium*, and included similar functional domain conservation to antiporters. Moreover in *E. ruminantium*, its homolog is classified as an integral membrane protein (Figure 8). It is unclear if the Ech_0379 gene product indeed represents a protein with antiporter function. Most bacterial genomes contain several genes encoding for antiporters that exchange Na^+^ and/or K^+^ for H^+^ from outside the cell [51]. On the contrary, intracellular pathogenic bacteria usually possess at most one antiporter. The *E. chaffeensis* genome, however, contains five homologs with obvious homology to antiporters (GenBank # CP000236.1). As the antiporters aid bacteria in meeting challenges of high or fluctuating pH, salt, temperature or osmolarity [51], they may represent an important set of proteins for an organism's intracellular survival *in vivo*. *E. chaffeensis* resides in a phagosome environment and most likely depends heavily on antiporters for its pH balance in a vertebrate host. Although we do not have any data supporting that Ech_0379 gene encodes an antiporter, the research reported in this study clearly demonstrates that it is an essential gene for the organism. Together, the predicted gene products of all three genes required for the pathogen's growth *in vivo* (Ech_0230, Ech_0379 and Ech_0660) appear to be associated with a membrane structure of *E. chaffeensis.*

This is the first study describing the targeted mutational methods for *Anaplasmataceae* pathogens. Similarly, this is the first in reporting the utility of transposon-based random mutagenesis in *Ehrlichia* species. Evidence was also presented for the first time in showing mutants impacting the growth of the organism in its natural host. Additional experimental manipulations are necessary for optimizing the protocols for recovering stable targeted mutants. A combination of the use of a targeted mutagenesis coupled with a method in expressing antisense RNA can aid in evaluating the importance of additional differentially expressed genes to the pathogen's adaptation to dual hosts and in causing pathogenesis. In particular, it is now possible in creating targeted mutations at a genomic location that does not impact the growth of the organism, such as the ones we identified in this study by random mutagenesis. Targeted insertions at such locations to synthesize complementary RNA of a gene of interest with an inducible promoter may be used to knockdown gene expression [52]–[54]. Creation of a large library of clones containing mutations in various genomic locations and assessing their importance to the pathogen's growth *in vivo* is feasible for bacteria having small genomes, such as *E. chaffeensis.* The molecular tools described in the current study, therefore, should aid in advancing our understanding of the role specific genes and genomic regions have in supporting the dual host life cycle of this and other related tick-borne pathogens.

## Materials and Methods

### *In vitro* cultivation of *E. chaffeensis*

*E. chaffeensis* (Arkansas isolate) was continuously cultivated in the canine macrophage cell line (DH82) essentially as described earlier [55]. The ISE6 and AAE2 cell lines originating from *Ixodes scapularis* and *A. americanum* ticks, respectively [56] were also used to cultivate *E. chaffeensis* by following the protocols reported earlier [16]. Detailed protocols for propagating the organisms were followed as described earlier [57].

### Selection of antibiotics for use in transformation experiments

*E. chaffeensis* was cultivated in T25 flasks having approximately 80% confluent DH82 cells. When the infectivity reached approximately 50%, cultures were detached from the flask using sterile glass beads and 0.5 ml each of the culture suspension was transferred to a 24 well plate. Spectinomycin, rifampin, gentamicin, chloramphenicol, kanamycin and ampicillin were tested for their ability to inhibit *E. chaffeensis* growth. The concentrations of the antibiotics tested were as follows: spectinomycin at 0, 10, 50,100,150 and 200 µg/ml; rifampin at 0, 0.1, 0.2 and 0.5 µg/ml; gentamicin at 0, 10, 20, 40, 60 and 80 µg/ml; chloramphenicol at 0, 2,4, 6, 8 and 10 µg/ml; Kanamycin at 0, 10, 20, 30, 40, 50 and 60 µg/ml. The assays were performed in triplicate wells for each antibiotic concentration assessed. *E. chaffeensis* infection levels in DH82 cultures were monitored once every three days for 20 days or longer by microscopic evaluation [52].

### DNA oligonucleotide primers

Various primers used in preparing the mutational constructs and for assessing the insertions as well as for analyzing gene expression are listed in Supplemental Table (Table S1). The primers were custom ordered from a commercial vendor (Integrated DNA Technologies, Inc., Coralville IA).

### Selection of genomic targets and preparation of constructs for creating targeted mutations by homologous recombination and TargeTron mutations

Based on RT-PCR analysis and cDNA microarray (Cheng et al. unpublished results and Kuriakose et al. [58]), some of the *E. chaffeensis* genes appeared to be non-expressed or expressed at low levels during growth of the organisms in macrophage or in tick cell cultures. Some of these genes were selected for creating targeted mutations by homologous recombination or a mobile group II intron based mutagenesis approach commonly referred to as the TargeTron method. Ech_0126, which appeared to be a transcriptionally silent gene, was chosen to build the homologous recombination constructs by two different formats (Rec I and Rec II). The Rec I construct was built by amplifying a 2.7 kb size fragment of the *E. chaffeensis* (Arkansas isolate) Ech_0126 gene spanning the genomic region from 113,771 to 116,518 (GenBank # CP000236.1). To promote directional cloning into the plasmid, primers were designed to contain restriction sites for Not I at the 5′ end and for Hind III at the 3′ end. The fragment was initially cloned into the PCR 2.1 Topo vector (Life Technologies, CA). The recombinant plasmid DNA was then digested with the Bstz17 I enzyme (which cuts at a site located1.07 kb from the 5′ end of the Ech_0126 insert), and used for inserting the antibiotic resistance cassette, CAT gene or the gentamicin resistance gene. [The antibiotic resistance genes were amplified from the plasmid pDEST10 (Life Technologies, CA).] To drive gene expression, the *rpsl* promoter, generated by PCR from the *E. chaffeensis* genome, was inserted upstream of the antibiotic resistance cassettes. The functionality of the *rpsl* promoter in driving the expression of the antibiotic resistance cassette genes was verified in *E. coli*. The *rpsl* promoter represents one of the highly active promoters in the *E. chaffeensis* genome (our unpublished results), possibly because it is responsible for driving the expression of several genes (GenBank # CP000236.1). A homolog of this promoter was also used earlier to drive expression of foreign genes in a related rickettsia, *Rickettsia prowazekii* [23], [24]. To create the Rec II construct, a 1.25 kb size 5′ fragment of the Ech_0126 gene (*E. chaffeensis* genome sequence coordinates: 113,771–115,024) and a 1.49 kb fragment at the 3′ end of the Ech_0126 gene (genome coordinates: 115,755–117,238) were amplified using *E. chaffeensis* genomic DNA as the template. PCR primers were designed to include a Not I site at the 5′ end and a Spe I site at the 3′ end for the 5′ end fragment, and Spe I site at the 5′ end and a Hind III site at the 3′ end for the 3′ end fragment to facilitate directional cloning into the plasmid vector. The PCR products were cloned into the PCR2.1 Topo vector by using the TA cloning method. The above described antibiotic resistance cassettes, including the *rpsl* promoter, were then cloned into the Spe I site. The inserts containing the *E. chaffeensis* Ech_0126 gene sequences and the antibiotic resistance cassette segments from the plasmid constructs were amplified using a proof reading enzyme, high fidelity platinum Taq DNA polymerase (Lifetechnologies, CA) to obtain linear fragments for use in homologous recombination experiments.

The genes Ech_0126, Ech_1136, and Ech_1143 and the intergenic regions between the genes Ech_0039 and Ech_0040, Ech_0111 and Ech_0112 and Ech_0251 and Ech_0252 were chosen for creating insertion mutations by employing the modified mobile group II intron-based TargeTron mutagenesis strategy (Sigma-Aldrich, St. Louis, MO). The TargeTron plasmid constructs were prepared by following the manufacturer's instructions. In particular, we used the web-based software program offered by the vendor in identifying sequences having the highest probability in introducing the modified mobile group II intron at the above identified *E. chaffeensis* genomic regions. Primer sets were designed to target each genomic site and used to modify the group II intron segment present in the plasmid construct, PACD4KC. In support of the expression of the group II intron in *E. chaffeensis*, the organism's *tuf* promoter (promoter for *E. chaffeensis* translation elongation factor gene, Ech_0407) was generated by PCR and cloned upstream of the modified group II intron segment by inserting at the Hind III restriction site. The activity of the *tuf* promoter was verified in *E. coli* for driving the expression of a promoterless β-galactosidase gene prior to its use in the *E. chaffeensis* experiments. To aid in selecting mutants at various genomic sites, the antibiotic resistance cassette, CAT gene, driven by the *E. chaffeensis rpsl* promoter was inserted at the Mlu II restriction enzyme site within the group II intron gene which replaced the built-in kanamycin resistance cassette.

### Plasmid constructs used for introducing random mutations

Himar1 A7 constructs used in this study were similar to the ones described earlier in [32] with minor modifications. Specifically, a single plasmid construct containing the Himar1 transposase gene driven by the *Anaplasma marginale* transcriptional regulator (Am-tr) promoter, and the transposon comprising a fluorescent protein marker gene and the spectinomycin/streptomycin antibiotic resistance gene (*aad*), also driven by the Am-tr promoter, was created. The transposon was flanked by the inverted repeats recognized by the transposase. Two different variations of the plasmids encoded either the green fluorescent protein (GFPuv) or the mCherry gene cloned upstream of the antibiotic resistance protein coding sequence. The plasmids are named pCis GFPuv-SS Himar A7 and pCis mCherry-SS Himar A7, respectively.

### Preparation of *E. chaffeensis* cultures for use in electroporation experiments

For targeted mutagenesis assays, *E. chaffeensis* infected DH82 cultures at about 80% infectivity were harvested from a confluent T25 flask, centrifuged at 2000×g for 10 min to collect the cell pellet which was then resuspended in 10 ml of 0.3 M sucrose solution. The solution was passed through 27.5 gauge needle 5 times to lyse the host cells. The cell lysate was then filtered through 2.7 and 1.6 µm filters (Millipore, Billerica, MA) and the solution was centrifuged at 15,500×g for 10 min to pellet the host cell-free organisms and the pellets were washed twice with 10 ml of ice-clod 0.3 M sucrose and resuspended in 50 µl of 0.3 M sucrose solution for use in transformation. *Ehrlichia* organisms are quantitated by performing the real-time PCR assay as we described earlier [59]. Although the number varies each time, typically we observe approximately 100 bacteria per each infected host cell.

For random mutagenesis using the Himar1 constructs, 3.5 ml of medium was carefully removed from one fully infected 5 ml ISE6 culture, and the cells were resuspended in the remaining medium. The suspension was transferred to a microfuge tube containing 0.2 ml of silicon carbide (#1 course rock tumbling grit, Loretone Inc., Mukilteo, WA), and vortexed for 30 sec at high speed. The supernatant was passed through a 2 µm pore-size filter (Whatman Ltd., Piscataway, NJ), and bacteria were collected by centrifugation at 11,000×g at 4°C for 5 min. Bacteria were washed twice in 0.3 M sucrose, and kept on ice between washes. Aliquots of purified *E. chaffeensis* (∼5×10^8^ per construct to be used) were resuspended in 50 µl of cold 0.3 M sucrose containing 1 µg of plasmid DNA and kept on ice for 15 min before electroporation (see below).

### Transformation experiments and culture evaluation

Six µg of purified linear PCR fragment from Rec I or Rec II constructs for homologous recombination or 6 µg of plasmid DNA for TargeTron or 1 µg of Himar1 plasmid DNA were mixed with 50 µl of host cell free *Ehrlichia* (containing about 2×10^9^ organisms for targeted mutagenesis or ∼5×10^8^ for transposon mutagensis) for each electroporation, transferred to 1 mm gap electroporation cuvettes, and incubated on ice for 15 min [32]. [Plasmid DNAs were prepared using a Maxiprep plasmid DNA isolation kit by following the manufacturer's instructions (Qiagen, CA).] *E. chaffeensis* organisms were electroporated at 2,000 volts, 25 µF and 400 Ω setting. The mixture was then combined with 0.5 ml of fetal bovine serum and 1 ml of DH82 or ISE6 cell suspension containing about 1×10^6^ cells. The sample was centrifuged at 5000×g for 5 min, incubated at room temperature for 15 min and then transferred into a sterile T25 flask with a confluent monolayer of DH82 or ISE6 cells. Cultures were incubated at 30°C overnight and then transferred to a 34°C incubator for ISE6 cells or 37°C incubator for DH82 cells for the continuous growth of the organisms. Three days later, an appropriate antibiotic with a desired concentration was added to the culture media in support of selecting the mutants containing the resistance gene against a specific antibiotic. The experiments with each targeted mutation construct were repeated at least three independent times. Himar1 mutants were selected in the presence of 100 µg/ml of spectinomycin and 100 µg/ml of streptomycin. The culture media containing antibiotics were replaced twice a week for DH82 cells and three times per week for ISE6 cells. When infectivity reached to 80% or higher, cell free *Ehrlichia* were prepared for inoculating a new flask of uninfected host cells with medium containing antibiotics. This procedure was repeated until all wild-type bacteria were eliminated. The presence of insertion mutations was monitored for 60 days or longer.

### Evaluation of mutations in *E. chaffeensis*

Genomic DNAs from the transformant flasks were extracted using the Puregene genomic DNA isolation kit as described by the manufacturer (Qiagen Inc., Valencia, CA). For targeted mutation analysis, PCR fragments amplified using a genome specific primer paired with an insertion specific primer from the 5′ end or 3′ end of integration region were amplified and the products were resolved on a 0.9% agarose gel, transferred to a nylon membrane and probed with an insertion specific probe. Once predicted size amplicons were identified, the products were purified and sequenced using the CEQ 8000 genetic analyzer (Beckman Coulter, CA). Sequences generated from this experiment were compared with the genomic region to identify the junctions.

For Himar1 transposon mutant analysis, 100 ng of genomic DNA recovered from the cultured organisms was digested with Spe I/Nde I, Bsrg I or EcoR V/Bgl II restriction enzymes for 3 hours at 37°C, resolved on a 0.9% agarose gel for about 6 hours at 60 volts, transferred to a nylon membrane by capillary transfer and then probed with a ^32^P labeled insertion specific probe at 68°C overnight by following standard molecular biology protocols [60]. The membrane was then exposed to an X-ray film to capture the radioactive signals from the hybridized DNA fragments. The genomic locations of the insertions were identified by inverse PCR or ST-PCR methods as described earlier [60], [61]. Briefly, for inverse PCR, 1 µg of genomic DNA from the mutant *Ehrlichia* cells were digested with Hind III, the restricted fragments were resolved on an agarose gel, specific genomic fragments were then purified and diluted in nuclease-free water; the DNA was ligated with T4 DNA ligase to favor the intramolecular ligation in a 400 µl volume. The ligated products were then used for inverse PCR [60] using insertion-specific primers designed to face away from each other on the genomic DNA strands. For ST-PCR, the initial PCR was performed using a random primer having a GATAT at the 3′ end and a degenerate sequence at the 5′ end paired with an insertion specific primer. The first PCR product was then purified and diluted to serve as the template in the nested PCR using a target specific nested primer and the second primer designed to prime to the degenerate sequence inserted in the first round of PCR by following the protocols described in [61].

### Genomic insertion region verification

A primer set was designed for use in the amplification of a segment from the genomic insertion sites; in this primer set, one primer was designed to bind to either 5′ or 3′ end of the insertion specific sequence and the second primer was designed to bind to the genomic region either upstream or downstream of the insertion site. The primers and the genomic DNAs isolated from the mutant cultures were used in the amplification assays and specific product amplification was verified by subjecting the PCR products to sequence analysis using a CEQ 8000 genetic analyzer (Beckman Coulter, CA).

### RNA isolation and RT-PCR

Total RNA from wild-type E. chaffeensis grown in DH82 or AAE2 cultures and Himar1 transformants grown in DH82 cultures was isolated by using Tri-reagent method following the manufacturer's instructions (Sigma-Aldrich, St. Louis, MO). Primers targeting to Ech_0379, Ech_0601 or Ech_0660 were designed for use in RT-PCR analysis. Total RNA was treated with RQ1 DNase at 37°C for 60 min to remove any genomic DNA contamination. For each set of RT-PCRs, one tube with reverse transcriptase, one tube without reverse transcriptase, one using DNA as the template and one with no template were included. For verifying RNA expression, the presence or absence of specific products in the assays containing RNA and reverse transcriptase was assessed and compared to the product generated from genomic DNA.

### Ethics statement

Animal experiments with deer were performed to comply with the Public Health Service (PHS) Policy on the Humane Care and Use of Laboratory Animals, the US Department of Agriculture's (USDA) Animal Welfare Act & Regulations (9CFR Chapter 1, 2.31), and with the prior approval of the Oklahoma State University (OSU) Institutional Animal Care and Use Committee (IACUC). Veterinary care for the animals was overseen by a University Veterinarian. At the end of each experiment, deer were euthanized and euthanasia was performed also in accordance with the university IACUC recommendations that are consistent with the recommendations of the Panel on Euthanasia of the American Veterinary Medical Association.

### Deer infections with mutant *E. chaffeensis*

Three day-old white-tailed deer fawns purchased from a breeder were reared in a tick free laboratory environment until the age of 3–5 months prior to utilizing them for experimental infection studies. Deer rearing and experimental infections were performed at Oklahoma State University (OSU) as per the guidelines outlined in the protocol. Deer were housed in a natural environment until utilized for infection studies. Fawns were moved to an Animal Biosafety Level facility for all experiments. Every effort is made to attend to the comfort and wellbeing of the deer. Food and water are provided ad libitum. During infection of the deer with *E. chaffeensis*, deer are housed in specially designed rubber-matted round enclosures twelve feet in diameter. Although confined to an enclosure, the deer were able to move around freely and were not restrained. Infected deer were monitored daily for signs of illness (decreased activity, decreased appetite, ruffed hair, nasal discharge, sneezing/coughing, etc.). Blood and serum samples were collected a maximum of two times per week and no more than 10 ml of blood was collected at each blood draw. We observed very mild to no symptoms associated with *E. chaffeensis* infections. At the end of each experiment, deer were euthanized as indicated above. Tissue harvests and animal disposal was done at the Oklahoma Animal Disease Diagnostic Laboratory.

*E. chaffeensis* mCherry transformant cultures at about 80–90% infectivity in T75 flask were harvested (about 15 ml), centrifuged at 15,000 g for 10 min at 4°C, supernatant was discarded and the culture was resuspended in 15 ml of 1×phosphate buffered saline (PBS). The washing steps were repeated twice and the final cell pellet was suspended in 7.5 ml of PBS to concentrate infected DH82 cells to about 2×10^6^ per ml (the estimated concentration of *Ehrlichia* organisms in the cells was approximately 2×10^8^ per ml). One ml each of the cell suspension was used for intravenous injections into a deer. The experiments were performed two independent times using freshly prepared cultures; the first experiment included one animal and the second experiment included two animals, all of which were inoculated with mCherry mutants. Deer infections were also performed similarly using the clonal population of mutants with mutation into the coding region of Ech_0660 (three deer) or intergenic region mutation into the non-coding region between the genes Ech_0284 and Ech_0285 (two deer).

### Evaluation of deer blood for infection by culture and PCR

About 3 ml of each of deer blood was collected in sterile EDTA tubes on day zero (prior to infection) and on days 3, 5, 6, 8, 10, 14, 18, 21, 28, 32, 35, 39, 42, 46, 49, 52, 56, 59, and 63 post-inoculation for the first experiment. Blood samples for the second infection were also collected on day zero and on 3, 7, 10, 13, 17, 20, 23, 30, 37 and 49 days post inoculation. The third infection blood samples were collected on day zero and on 3, 6, 9, 13, 16, and 20 days post inoculation. The blood samples were stored at 4°C until use (maximum of three days). Blood samples were spun at 3,000 rpm in a Clay Adams Sero-fuge (Becton Dickinson, Sparks, MD) for 5 min. Plasma was removed and about 1 ml of buffy coat each was transferred to a 15 ml sterile Falcon centrifuge tube containing 10 ml RBC lysis buffer (155 mM NH_4_Cl, 10 mM KHCO_3_ and 0.1 mM EDTA) and mixed several times until complete lysis of erythrocytes. The samples were then centrifuged at 5,000 g for 5 min and the supernatants were discarded. The buffy coat pellet from each sample was resuspended in 300 µl of 1×PBS. To assess for infection with *E. chaffeensis*, 100 µl each of the cell suspensions was transferred into a well of 12-well sterile culture plate containing 0.9 ml of DH82 cells having about 80% confluency. The cultures were grown by following the detailed culture protocols reported earlier [57] and infection was monitored twice a week by examining the Hema3 stained cytospin slides for up to 8–10 weeks to determine if a sample was positive or negative for the organisms. Contents of positive infection were transferred to a T25 flask and allowed to grow until infection reached about 80% and were used for genomic DNA isolation or for liquid nitrogen storage.

One hundred µl each of the buffy coats from deer blood were also used for isolating total genomic DNA by using the Wizard SV Genomic DNA purification kit as per the manufacturer's instructions (Promega, Madison, WI); purified DNA from each sample was stored in 100 µl of buffer containing 10 mM Tris-HCl and 1 mM EDTA (pH 8.0) (TE buffer). The DNAs were used to assess *E. chaffeensis* infection status by performing semi-nested PCR targeting the insertion specific spectinomycin resistance gene (*aad*) (primers for this experiment are listed in Table S1). Briefly, 2 µl of genomic DNA from deer blood was used for the first round PCR in a 25 µl reaction volume using Platinum Taq DNA polymerase as per the manufacturer's instructions (Life Technologies, Grand Island, NY). The PCRs were performed in a GenAmp9700 instrument (Applied Biosystems, Foster City, CA) with the following temperature cycles: 94°C for 4 min, followed by 35 cycles of 94°C for 30 s, 52°C for 30 s, and 72°C for 1 min and 1 cycle of 72°C for 3 min. The nested PCR was done using the same PCR conditions as the first round PCR and the templates for the second round included 2 µl of 1∶100 diluted products from the first PCR and with nested PCR primer set. Samples that were positive for the *aad* gene were also evaluated by nested PCRs targeting the transposon insertions and flanking genomic regions in the five mCherry mutants defined (primers for this experiment are listed in Table S1).

### Tick attachment

Nymphal *A. americanum* ticks were obtained from the National Tick Research and Education Resource (NTRER) at Oklahoma State University. Laboratory-reared ticks were propagated following the published protocols of Patrick and Hair [62]; and in accordance with the approval from the IACUC and as per the guidelines outlined in the approved protocol. About 300 nymphal *A. americanum* ticks were placed on infected deer starting day 5 post inoculation. Engorged ticks were collected after blood meal (typically in about 7 days) and stored at room temperature and 96% humidity chamber and permitted to molt to adults (took approximately 45–50 days). Genomic DNA was isolated from individual adult ticks by using the Wizard SV Genomic DNA purification kit as outlined above. Final purified DNAs were resuspended in 100 µl each of TE buffer. Two µl each was then used for nested PCR analysis targeting to the *aad* gene or targeting to the transposon insertion regions as described above.

## Supporting Information

Figure S1 **An illustration outlining the strategy used in creating the constructs for homologous recombination.** The *E. chaffeensis* genomic region selected for the homologous recombination is presented at the top of the cartoon. The solid big black arrow in the middle at the top panel represents the gene, Ech_0126, and its orientation in the genome and the big black arrows on the left and right represent genomic regions representing genes Ech_0125 and Ech_0127, respectively. The small arrows in the figure represent location of primers designed for construct preparation and for assessing the insertions following the homologous recombination (detailed list of the primers is included in Table S1). The PCR products section represents the segments amplified from the *E. chaffeensis* genome for Rec I and Rec II constructs preparation. The solid red arrows represent the antibiotic resistance gene coding regions used for the experiment (CAT) and the solid blue bars represent the *E. chaffeensis rpsl* promoter segments inserted for driving the expression of antibiotic resistance genes.
(TIF)

Figure S2 **Southern blot analysis of homologous recombination constructs assessed following transformation into** ***E. chaffeensis*** **.** PCR products generated from *E. chaffeensis* genomic DNA recovered after different time points post electroporation of Rec I or Rec II segments for homologous recombination were assessed by DNA blot analysis. The primers used for the amplification included forward primer targeting to the genomic region upstream of the insertion and the reverse primer targeting to the antibiotic cassette. Predicted size products for Rec I and RecII recombination (1.86 kb and 1.96 kb, respectively) were detected for different times in culture post electroporation. Panels A and B represent the data from two independent experiments. Numbers above the lanes in panel A refer to hours post transformation, whereas numbers in panel B represent days post transformation. A non-transformed *E. chaffeensis* genomic DNA control was included in the far right lane in panel B (Ec). To define the specificity of the amplified products, PCR products resolved on an agarose gel and transferred to a nylon membrane were hybridized using a ^32^p labeled antibiotic resistance gene-specific probe. Further, the product integrity was verified by PCR DNA sequence analysis.
(TIF)

Figure S3 **Southern blot analysis of TargeTron constructs assessed following transformation into** ***E. chaffeensis*** **.** PCR products generated from *E. chaffeensis* genomic DNA recovered after different time points post electroporation and culture growth of the TargeTron constructs of Ech_0126, Ech_1136, and Ech_1143 genes and for non-coding region spanning between Ech_0111 and Ech_0112. The primers used for the amplification included a forward primer targeting to the genomic region upstream to the insertion site and a reverse primer targeting to the mobile group II intron gene region. Predicted size products are 2811 bp, 891 bp, 399 bp and 2,139 bp for the genes Ech_0126, Ech_1136, and Ech_1143 and for the non-coding region spanning between Ech_0111 and Ech_0112, respectively. The predicted size products were observed only for the genes Ech_0126 (lane 1) and Ech_1143 (lane 3) and for the non-coding region between Ech_0111 and Ech_0112 genes (lane 4), but not for Ech_1136 (lane 2). Similar analysis for the intergenic regions between the genes of Ech_0039 and Ech_0040 and Ech_0251 and Ech_0252 also did not result in insertions as judged by Southern blot analysis (not included in this Figure). This experiment was repeated three independent times and data from one of the experiments were presented here. To define the specificity of the amplicons, the PCR products resolved on an agarose gel were hybridized using a ^32^p labeled group II-intron-specific probe.
(TIF)

Table S1 **List of oligonucleotides used in this study.** The primers in the list include those used for preparing the constructs for homologous recombination, TargeTron constructs and their associated primer sets used for screening the insertions into the genome. The primer sets also included those used for preparing the promoter segments and antibiotic resistance cassettes inserted in the constructs. Primers used for screening the transposon insertions into the genome as well as those used for screening the gene expressions described in the manuscript were also listed in the table. *E. chaffeensis* genes for which the primers targeted are identified by their gene identification numbers listed in the whole genome sequence.
(DOCX)
