# Correction: Targeted and Random Mutagenesis of *Ehrlichia chaffeensis* for the Identification of Genes Required for *In vivo* Infection

**DOI:** 10.1371/annotation/322c8bba-8dae-4196-b85d-5de315e44d76

**Published:** 2013-05-21

**Authors:** Chuanmin Cheng, Arathy D. S. Nair, Vijaya V. Indukuri, Shanzhong Gong, Roderick F. Felsheim, Deborah Jaworski, Ulrike G. Munderloh, Roman R. Ganta

The last row of Table 2 is missing. Please see the corrected Table 2 here: http://www.plospathogens.org/corrections/ppat.1003171.t002.cn.docx

