# Supplementary figures and images for: Targeted and Random Mutagenesis of Ehrlichia chaffeensis for the Identification of Genes Required for In vivo Infection
Source: PLoS Pathog. 2013 Feb 14;9(2):e1003171. doi: 10.1371/journal.ppat.1003171 (PMC3573109; doi:10.1371/journal.ppat.1003171)

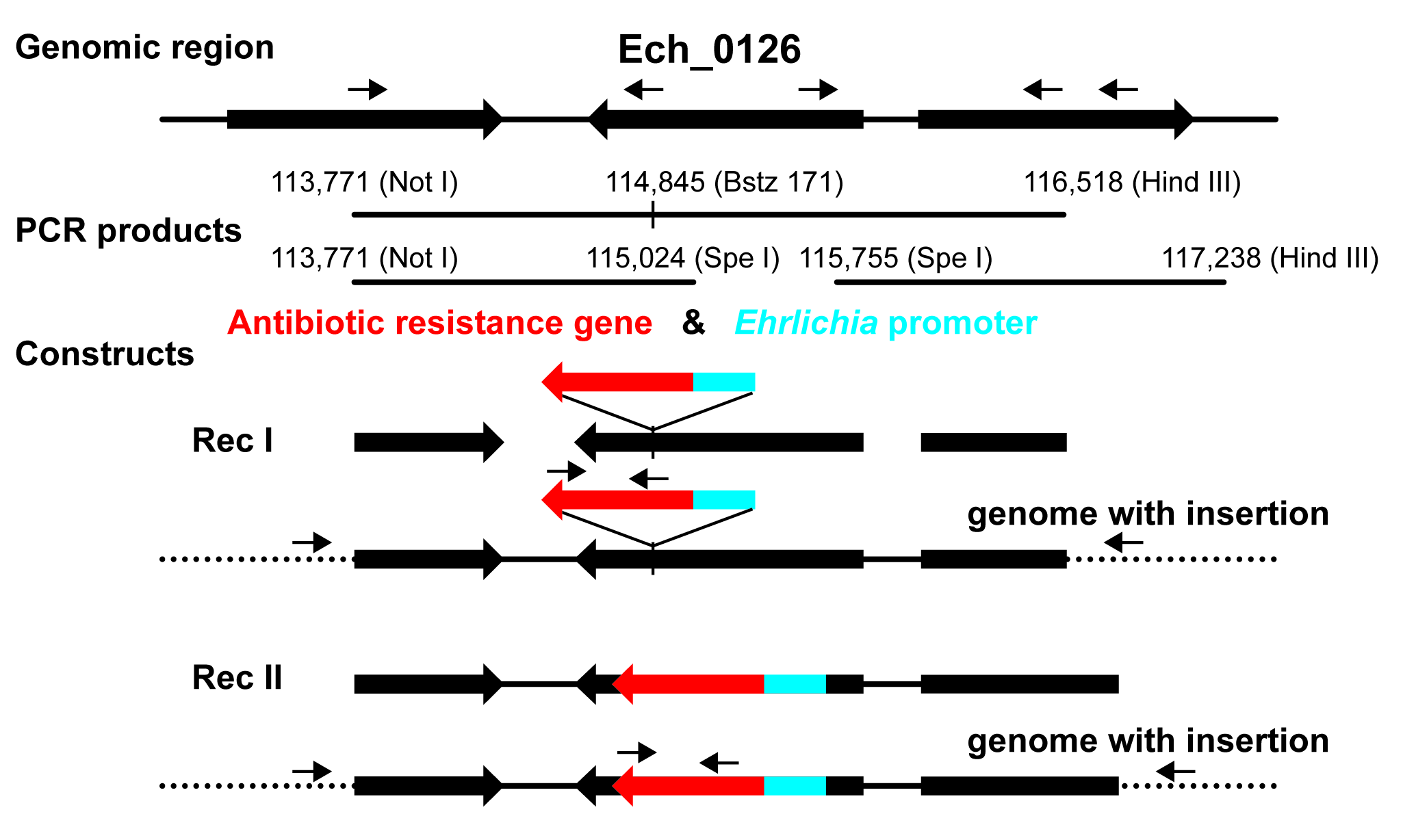

Supplement: Figure S1 — An illustration outlining the strategy used in creating the constructs for homologous recombination. The E. chaffeensis genomic region selected for the homologous recombination is presented at the top of the cartoon. The solid big black arrow in the middle at the top panel represents the gene, Ech_0126, and its orientation in the genome and the big black arrows on the left and right represent genomic regions representing genes Ech_0125 and Ech_0127, respectively. The small arrows in the figure represent location of primers designed for construct preparation and for assessing the insertions following the homologous recombination (detailed list of the primers is included in Table S1). The PCR products section represents the segments amplified from the E. chaffeensis genome for Rec I and Rec II constructs preparation. The solid red arrows represent the antibiotic resistance gene coding regions used for the experiment (CAT) and the solid blue bars represent the E. chaffeensis rpsl promoter segments inserted for driving the expression of antibiotic resistance genes. (TIF) [file ppat.1003171.s001.tif]

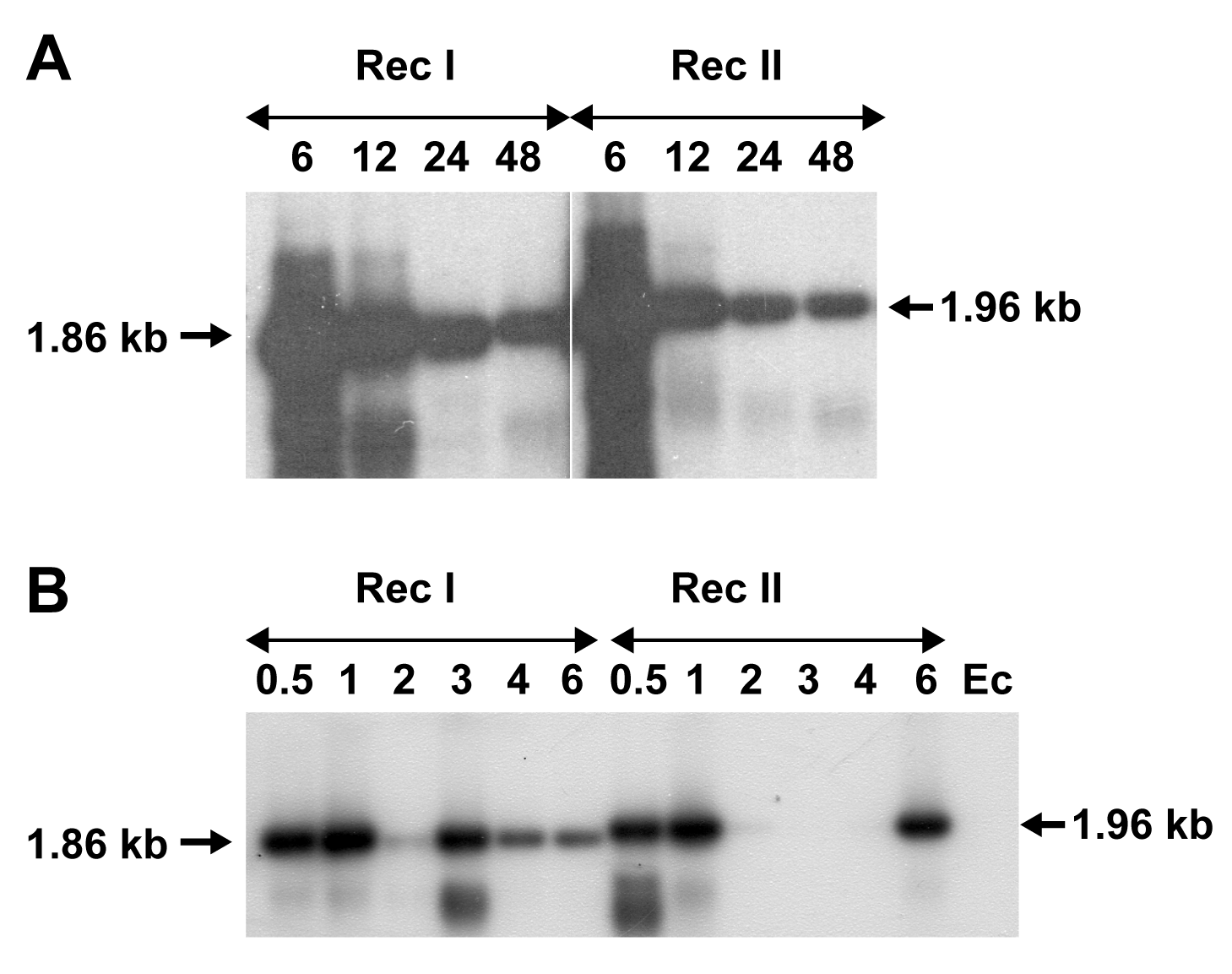

Supplement: Figure S2 — Southern blot analysis of homologous recombination constructs assessed following transformation into E. chaffeensis . PCR products generated from E. chaffeensis genomic DNA recovered after different time points post electroporation of Rec I or Rec II segments for homologous recombination were assessed by DNA blot analysis. The primers used for the amplification included forward primer targeting to the genomic region upstream of the insertion and the reverse primer targeting to the antibiotic cassette. Predicted size products for Rec I and RecII recombination (1.86 kb and 1.96 kb, respectively) were detected for different times in culture post electroporation. Panels A and B represent the data from two independent experiments. Numbers above the lanes in panel A refer to hours post transformation, whereas numbers in panel B represent days post transformation. A non-transformed E. chaffeensis genomic DNA control was included in the far right lane in panel B (Ec). To define the specificity of the amplified products, PCR products resolved on an agarose gel and transferred to a nylon membrane were hybridized using a 32p labeled antibiotic resistance gene-specific probe. Further, the product integrity was verified by PCR DNA sequence analysis. (TIF) [file ppat.1003171.s002.tif]

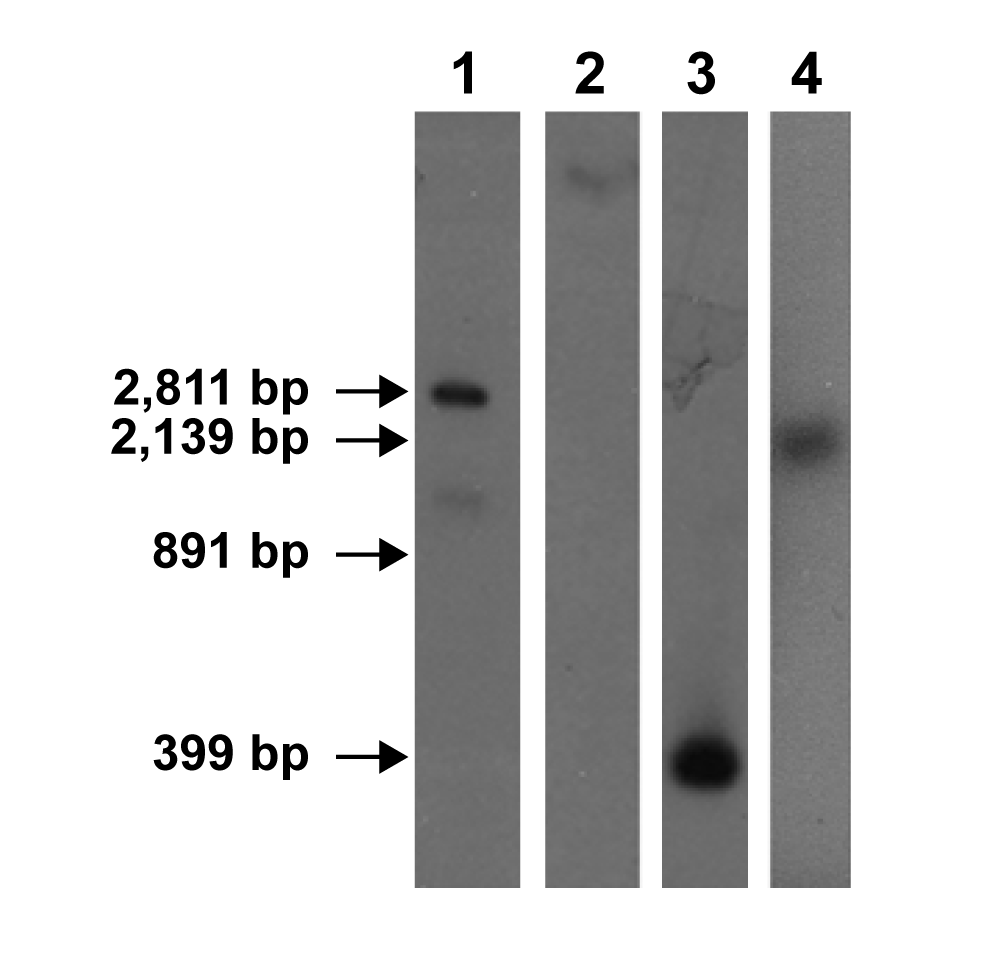

Supplement: Figure S3 — Southern blot analysis of TargeTron constructs assessed following transformation into E. chaffeensis . PCR products generated from E. chaffeensis genomic DNA recovered after different time points post electroporation and culture growth of the TargeTron constructs of Ech_0126, Ech_1136, and Ech_1143 genes and for non-coding region spanning between Ech_0111 and Ech_0112. The primers used for the amplification included a forward primer targeting to the genomic region upstream to the insertion site and a reverse primer targeting to the mobile group II intron gene region. Predicted size products are 2811 bp, 891 bp, 399 bp and 2,139 bp for the genes Ech_0126, Ech_1136, and Ech_1143 and for the non-coding region spanning between Ech_0111 and Ech_0112, respectively. The predicted size products were observed only for the genes Ech_0126 (lane 1) and Ech_1143 (lane 3) and for the non-coding region between Ech_0111 and Ech_0112 genes (lane 4), but not for Ech_1136 (lane 2). Similar analysis for the intergenic regions between the genes of Ech_0039 and Ech_0040 and Ech_0251 and Ech_0252 also did not result in insertions as judged by Southern blot analysis (not included in this Figure). This experiment was repeated three independent times and data from one of the experiments were presented here. To define the specificity of the amplicons, the PCR products resolved on an agarose gel were hybridized using a 32p labeled group II-intron-specific probe. (TIF) [file ppat.1003171.s003.tif]
