# Supplementary material for: Targeted and Random Mutagenesis of Ehrlichia chaffeensis for the Identification of Genes Required for In vivo Infection
Source: PLoS Pathog. 2013 Feb 14;9(2):e1003171. doi: 10.1371/journal.ppat.1003171 (PMC3573109; doi:10.1371/journal.ppat.1003171)
Supplement: Table S1 — List of oligonucleotides used in this study. The primers in the list include those used for preparing the constructs for homologous recombination, TargeTron constructs and their associated primer sets used for screening the insertions into the genome. The primer sets also included those used for preparing the promoter segments and antibiotic resistance cassettes inserted in the constructs. Primers used for screening the transposon insertions into the genome as well as those used for screening the gene expressions described in the manuscript were also listed in the table. E. chaffeensis genes for which the primers targeted are identified by their gene identification numbers listed in the whole genome sequence. (DOCX) [file ppat.1003171.s004.docx]

| **Supplemental Table 1:** List of oligonucleotides used in this study | | | |  | |  | |
| --- | --- | --- | --- | --- | --- | --- | --- |
|  | |  | |  | |  | |
| **Primer*** | | **Sequence** | | **Orientation** | | **Size (bp)** | |
|  | |  | |  | |  | |
| **HOMOLOGOUS RECOMBINATION** | |  | | |  | |  |
| **Constructs preparation** | |  | |  | |  | |
| **Rec I** | |  | |  | |  | |
| RRG650 | | 5'acgt*gcggccgc*gggattcctgaagtactacaagg | | forward | | 2747 | |
| RRG651 | | 5'acgt*aagctt*catgacagacataactacaattaacg | | reverse | |  | |
|  | |  | |  | |  | |
| **Rec II** | |  | |  | |  | |
| RRG650 | |  | | forward | | 1253 | |
| RRG652 | | 5'aaaa*actagt*agagatagaaatacag | | reverse | |  | |
|  | |  | |  | |  | |
| RRG653 | | 5'acgt*actagt*ttactgtagaaaagtcaatgttg | | forward | | 1483 | |
| RRG654 | | 5'agac*aagctt*gaaataatattaaatgttg | | reverse | |  | |
|  | |  | |  | |  | |
| **Antibiotic cassette** | |  | |  | |  | |
| rpsl promoter | |  | |  | |  | |
| Rpsl-For | | 5'gat*acgcgt*tagattattttgaaaggaatgatttc | | forward | | 293 | |
| rpsl-Rev | | 5'gat*catatg*atgaatgtaataaccttacag | | reverse | |  | |
|  | |  | |  | |  | |
| Chloramphenicol acetyl transferase | | | |  | |  | |
| Cat-For | | 5'acaa *catatg*atggagaaaaaaatcactggatatacc | | forward | | 660 | |
| RRG723 | | 5'gtc*acgcgt*ttacgccccgccctgcca | | reverse | |  | |
|  | |  | |  | |  | |
| **Insertion screening** | |  | |  | |  | |
| RRG784 | | 5'tgtacttcgtgctggatgtagg | | forward | | 1824 (Rec I) | |
| RRG833 | | 5'actactgttgatattagtcaatggc | | reverse | | 2003 (Rec II) | |
|  | |  | |  | |  | |
| **TARGETRON CONSTRUCTS** |  | |  | |  | | |
| **constructs preparation** | |  | |  | |  | |
| **Ech_0126** | |  | |  | |  | |
| 260\|261s-IBS | | 5'aaaaaaagcttataattatccttaagctactggagagtgcgcccagatagggtg | | forward | | 350 | |
| 260\|261s-EBS1d | | 5'cagattgtacaaatgtggtgataacagataagtctggagaaataacttacctttctttgt | | reverse | |  | |
| 260\|261s-EBS2 | | 5'tgaacgcaagtttctaatttcgatttagcttcgatagaggaaagtgtct | | forward | |  | |
| EBS universal | | 5'cgaaattagaaacttgcgttcagtaaac | | reverse | |  | |
|  | |  | |  | |  | |
| **Ech_1136** | |  | |  | |  | |
| 174\|175a-IBS | | 5'aaaaaagcttataattatccttattcttcagctgagtgcgcccagatagggtg | | forward | | 350 | |
| 174\|175a-EBS1d | | 5'cagattgtacaaatgtggtgataacagataagtcagctgagataacttacctttctttgt | | reverse | |  | |
| 174\|175a-EBS2 | | 5'tgaacgcaagtttctaatttcggttaagaatcgatagaggaaagtgtct | | forward | |  | |
| EBS universal | |  | | reverse | |  | |
|  | |  | |  | |  | |
| **Ech_1143** | |  | |  | |  | |
| 351\|352s-IBS | | 5'aaaaaagcttataattatccttaagaatcgagcttgtgcgcccagatagggtg | | forward | | 350 | |
| 351\|352s-EBS1d | | 5'cagattgtacaaatgtggtgataacagataagtcgagcttgataacttacctttctttgt | | reverse | |  | |
| 351\|352s-EBS2 | | 5'tgaacgcaagtttctaatttcgattattcttcgatagaggaaagtgtct | | forward | |  | |
| EBS universal | |  | | reverse | |  | |
|  | |  | |  | |  | |
| **Ech_0039-Ech_0040** | |  | |  | |  | |
| RRG727(683\|684s-IBS) | | 5'aaaaaagcttataattatccttaatggcctggttggtgcgcccagatagggtg | | forward | | 350 | |
| RRG728(683\|684s-EBS1d ) | | 5'cagattgtacaaatgtggtgataacagataagtctggttgggtaacttacctttctttgt | | reverse | |  | |
| RRG729(683\|684s-EBS2 ) | | 5'tgaacgcaagtttctaatttcggttgccatccgatagaggaaagtgtct | | forward | |  | |
| EBS universal | |  | | reverse | |  | |
|  | |  | |  | |  | |
| **Ech_0111-Ech_0112** | |  | |  | |  | |
| RRG730(487\|488a-IBS) | | 5'aaaaaagcttataattatccttatgaaccaccaaagtgcgcccagatagggtg | | forward | | 350 | |
| RRG731(487\|488a-EBS1d) | | 5'cagattgtacaaatgtggtgataacagataagtcaccaaacctaacttacctttctttgt | | reverse | |  | |
| RRG732(487\|488a-EBS2) | | 5'tgaacgcaagtttctaatttcggttgttcatcgatagaggaaagtgtct | | forward | |  | |
| EBS universal | |  | | reverse | |  | |
|  | |  | |  | |  | |
| **Ech_0251-Ech_0252** | |  | |  | |  | |
| RRRG733(698\|699s-IBS) | | 5'aaaaaagctataattatccttaaaggacatctgcgtgcgcccagatagggtg | | forward | | 350 | |
| RRG734(698\|699s-EBS1d) | | 5'cagattgtacaaatgtggtgataacagataagtcatctgctttaacttacctttctttgt | | reverse | |  | |
| RRG735(698\|699s-EBS2) | | 5'tgaacgcaagtttctaatttcgatttcctttcgatagaggaaagtgtct | | forward | |  | |
| EBS universal | |  | | reverse | |  | |
|  | |  | |  | |  | |
| **Tuf promoter** | |  | |  | |  | |
| RRG725 | | 5'gtc*aagctt*aaaaatgtgactattaattttgac | | forward | | 367 | |
| RRG726 | | 5'gtc*aagcttaag*aaacaaatacctttaacatc | | reverse | |  | |
|  | |  | |  | |  | |
| **insertion screening** | |  | |  | |  | |
| **Ech-0126** | |  | |  | |  | |
| EBS universal | |  | | forward | | 2811 | |
| RRG650 | |  | | reverse | |  | |
|  | |  | |  | |  | |
| **Ech_1136** | |  | |  | |  | |
| EBS universal | |  | | forward | | 819 | |
| RRG71 | | 5'gagctccttctaatactac | | reverse | |  | |
|  | |  | |  | |  | |
| **Ech_1143** | |  | |  | |  | |
| EBS universal | |  | | forward | | 399 | |
| RRG34 | | 5'gaagcgcaatatccaactcctc | | reverse | |  | |
|  | |  | |  | |  | |
| **Ech_0039-Ech_0040** | |  | |  | |  | |
| EBS universal | |  | | forward | | 290 | |
| RRG736 | | 5’gtgctatcctgcgctgtaatatg | | reverse | |  | |
|  | |  | |  | |  | |
| **Ech_0111-Ech_0112** | |  | |  | |  | |
| RRG739 | | 5’cagtaaaaccatagtgcgtcgatac | | forward | | 370 | |
| EBS universal | |  | | reverse | |  | |
|  | |  | |  | |  | |
| **Ech_0251-Ech_0252** | |  | |  | |  | |
| EBS universal | |  | | forward | | 289 | |
| RRG740 | | 5’tctgttacaatgatggatattaag | | reverse | |  | |
|  | |  | |  | |  | |
|  | |  | |  | |  | |
| **TRANSPOSON MUTATION** | |  | |  | |  | |
| **Mutation screening** | |  | |  | |  | |
| **Ech_0231-Ech_0232** | |  | |  | |  | |
| RRG1254 | | 5'gtggattgcttataggagcaatagg | | forward | | 785 | |
| RRG1200 | | 5'gttacggtgaccgtaaggcttg | | reverse | |  | |
|  | |  | |  | |  | |
| RRG1254 | |  | | forward | | 231 | |
| RRG1202 | | 5'cagttggaagaatttgttcactacgt | | reverse | |  | |
|  | |  | |  | |  | |
| **Ech_0284-Ech_0285** | |  | |  | |  | |
| RRG1225 | | 5'ttagtgggcttgatgcaggac | | forward | | 913 | |
| RRG1200 | |  | | reverse | |  | |
|  | |  | |  | |  | |
| RRG1225 | |  | | forward | | 335 | |
| RRG1202 | |  | | reverse | |  | |
|  | |  | |  | |  | |
| **Ech_0379** | |  | |  | |  | |
| RRG1276 | | 5'ctaaggttgtagggaatgcaacc | | forward | | 997 | |
| RRG1200 | |  | | reverse | |  | |
|  | |  | |  | |  | |
| RRG1276 | |  | | forward | | 419 | |
| RRG1202 | |  | | reverse | |  | |
|  | |  | |  | |  | |
| **Ech_0479-Ech_0480** | |  | |  | |  | |
| RRG1223 | | 5'gatgatggtgcttttagtaggtatg | | forward | | 386 | |
| RRG1194 | | 5'tatcccttatgttactcagataacttagg | | reverse | |  | |
|  | |  | |  | |  | |
| **Ech_0490-Ech_0492** | |  | |  | |  | |
| RRG1278 | | 5'tcgtgctgctatgttcatgcatg | | forward | | 464 | |
| RRG1194 | |  | | reverse | |  | |
|  | |  | |  | |  | |
| **Ech_0202 and Ech_0203** | |  | |  | |  | |
| RRG1256 | | 5'agaggagcatgttcagggtgtcc | | forward | | 878 | |
| RRG1200 | |  | | reverse | |  | |
|  | |  | |  | |  | |
| RRG1256 | |  | | forward | | 300 | |
| RRG1202 | |  | | reverse | |  | |
|  | |  | |  | |  | |
| **Ech_0601** | |  | |  | |  | |
| RRG1227 | | 5'acacctaatggattttcaacaagatg | | forward | | 770 | |
| RRG1200 | |  | | reverse | |  | |
|  | |  | |  | |  | |
| RRG1227 | |  | | forward | | 202 | |
| RRG1202 | |  | | reverse | |  | |
|  | |  | |  | |  | |
| **Ech_0660** | |  | |  | |  | |
| RRG1344 | | 5'tgtaactgtatcctcacctatcacc | | forward | | 437 | |
| RRG1194 | |  | | reverse | |  | |
|  | |  | |  | |  | |
| **Ech_0760-Ech_0761** | |  | |  | |  | |
| RRG1280 | | 5'agtcgtgcaagaaagcttagagg | | forward | | 592 | |
| RRG1194 | |  | | reverse | |  | |
|  | |  | |  | |  | |
| **RT-PCR primers** | |  | |  | |  | |
| **Ech_0601** | |  | |  | |  | |
| RRG1227 | |  | | forward | | 187 | |
| RRG1228 | | 5'gagaatactgtttatgtgtcagtgac | | reverse | |  | |
|  | |  | |  | |  | |
| **Ech_0379** | |  | |  | |  | |
| RRG1282 | | 5’tgaaaa tctg atcgatagtg ctgtgg | | forward | | 376 | |
| RRG1283 | | 5'ggttgcattccctacaaccttag | | reverse | |  | |
|  | |  | |  | |  | |
| **Ech_0660** | |  | |  | |  | |
| RRG1344 | |  | | forward | | 265 | |
| RRG1345 | | 5'ctatcaattcttcacttccatttgtgtg | | reverse | |  | |
|  | |  | |  | |  | |
| **ST-PCR PRIMERS** | |  | |  | |  | |
| First round | |  | |  | |  | |
| RRG1260 | | 5'ggccacgcgtcgactagtac(N)_10_gatat | | forward | | Varies | |
| RRG1194 | |  | | reverse | |  | |
|  | |  | |  | |  | |
| Second round | |  | |  | |  | |
| RRG1261 | | 5'ggccacgcgtcgactagtac | | forward | | Varies | |
| RRG1258 | | 5'tgcaacagttatttaatgtatggttg | | reverse | |  | |

*Sequence for the primers was provided only once if a primer is listed multiple times.
